# Neotropical bee microbiomes point to a fragmented social core and strong species-level effects

**DOI:** 10.1186/s40168-023-01593-z

**Published:** 2023-07-14

**Authors:** Jordan G. Kueneman, Ernesto Bonadies, Devin Thomas, David W. Roubik, William T. Wcislo

**Affiliations:** 1 Smithsonian Tropical Research Institute, Panama City, Panama; 2 Department of Entomology, Cornell University, Comstock Hall, 2126, Ithaca, NY 14853 Czech Republic; 3 Biology Centre of the Czech Academy of Sciences, Branisovska, České Budějovice, Czech Republic; 4 University of New Hampshire, Durham, NH USA

## Abstract

**Background:**

Individuals that band together create new ecological opportunities for microorganisms. In vertical transmission, theory predicts a conserved microbiota within lineages, especially social bees. Bees exhibit solitary to social behavior among and/or within species, while life cycles can be annual or perennial. Bee nests may be used over generations or only once, and foraging ecology varies widely. To assess which traits are associated with bee microbiomes, we analyzed microbial diversity within solitary and social bees of Apidae, Colletidae, and Halictidae, three bee families in Panama’s tropical forests. Our analysis considered the microbiome of adult gut contents replicated through time, localities, and seasons (wet and dry) and included bee morphology and comparison to abdominal (dissected) microbiota. Diversity and distribution of tropical bee microbes (TBM) within the corbiculate bee clade were emphasized.

**Results:**

We found the eusocial corbiculate bees tended to possess a more conserved gut microbiome, attributable to vertical transmission, but microbial composition varied among closely related species. Euglossine bees (or orchid bees), corbiculates with mainly solitary behavior, had more variable gut microbiomes. Their shorter-tongued and highly seasonal species displayed greater diversity, attributable to flower-visiting habits. Surprisingly, many stingless bees, the oldest corbiculate clade, lacked bacterial genera thought to predate eusociality, while several facultatively social, and solitary bee species possessed those bacterial taxa. Indeed, nearly all bee species displayed a range of affinities for single or multiple variants of the “socially associated” bacterial taxa, which unexpectedly demonstrated high sequence variation.

**Conclusions:**

Taken together, these results call into question whether specific bacterial associates facilitate eusocial behavior, or are subsequently adopted, or indicate frequent horizontal transmission between perennial eusocial colonies and other social, facultatively social, and solitary bees.

Video Abstract

**Supplementary Information:**

The online version contains supplementary material available at 10.1186/s40168-023-01593-z.

## Introduction

Sociality is a major evolutionary innovation that represents a unique level of biological organization [1]. In many habitats, eusocial bees are an ecologically dominant species among pollinators and bees. They include colonies of honey bees (Apini, Apidae), bumble bees (Bombini, Apidae), stingless bees (Meliponini, Apidae), and certain sweat bees (Halictinae). Yet approximately 90% of all bees are solitary or parasitic [2, 3], which raises questions about the relative advantages of social living. Here, we further query the influence of pathogenic or beneficial microbes in shaping social behavior, or bee abundance and success. Stingless bee and honey bee colonies are perennial, while nearly all other bee colonies and all solitary bees are not (for natural history details, see Supplemental Materials, S1). A perennial nest environment favors pathogen transmission [4], but also enables co-evolution between hosts and their beneficial microbiomes due to vertical transmission [5–7]. How annual colonies and univoltine or multivoltine bees mitigate pathogens and the degrees to which they maintain beneficial microbial associations are currently unknown for most species.

Bees interact with coevolved bacteria, fungi, microeukaryotes, and viruses [8]. Certain microbes prevent provisioned nutrients from spoiling, and others permit dietary processing through pollen degradation and detoxification [9–12]. For example, a solitary bee (*Osmia*) deprived of microbes has reduced growth and survivorship [13, 14] and a highly eusocial bee species (*Scaptotrigona*) needs to consume mutualistic fungi to pupate [15, 16]. Bee-associated microbes also mediate immune functions and defend against diverse pathogens [17–20]. Thus, increasing evidence shows that microbiota affect bee health and fitness in numerous ways, but our knowledge is still quite limited.

The family Apidae includes a clade of corbiculate bees that transport pollen in baskets or “corbiculae” (tribes Apini, Bombini, Meliponini, and Euglossini). Those more than 1000 species display considerable variation in degree of sociality [21]. Available data indicate that specialized microbes occur in the adult bee midgut, ilium, and rectum, and they include *Gilliamella*, *Snodgrassella*, *Lactobacillus*, and *Bifidobacterium* [20]. Variation in the presence of these derived microbial taxa occurs among stingless bees and bumble bees [7, 22], and several additional bacterial taxa are thought to have coevolved with only one bee tribe [23]. However, the microbiomes of many corbiculate taxa are unknown, including the entire tribe Euglossini, which includes 250 or more solitary, weakly social, or parasitic species [24, 25].

Bee microbiomes are initially established in one of two ways. In perennial colonies, individuals are inoculated upon adult emergence, after ingested food is voided. Those social bees support microbial colonization which occurs through oral trophallaxis, or contact with feces and nurse bees, transmitted vertically [26]. Solitary and short-lived bees never or rarely contact their offspring [27]. Their microbiomes originate from the pollen and nectar provided by the mother and are environmentally acquired during her foraging. They are horizontally transmitted from flowers [22].

Our study evaluates the microbiome of biologically diverse bees, containing highly social to completely solitary species within Apini, Bombini, Meliponini, Euglossini, Caupolicanini, Augochlorini, and Halictini in the Republic of Panama, which includes ~70,000 km^2^ and distinct forest types and environmental conditions (e.g., rainfall can vary 10-fold over relatively small distances [28]). By investigating gut symbiont data in a relatively constrained geographic space, through time, we sought to improve the understanding of microbial associations within bee clades and life history strategies (emphasizing vertical or horizontal microbial transmission), while including location, seasonality, and morphological traits as other variables. Our approach largely addresses the phylogenetic and/or geographic limitations of previous studies [15, 29, 30] while offering novel analyses of the effects of seasonality and host morphology on bee microbiomes. As we examine the dynamics and potential origins of microbial diversity in bee biology, we also tested the assumption that a “core corbiculate gut microbiota” (referred to as CCGM) exists among corbiculate bees and assess whether these microbes are more widely distributed across bee taxa.

## Materials/subjects and methods

### Bee sampling and processing

**Fig. 1:**
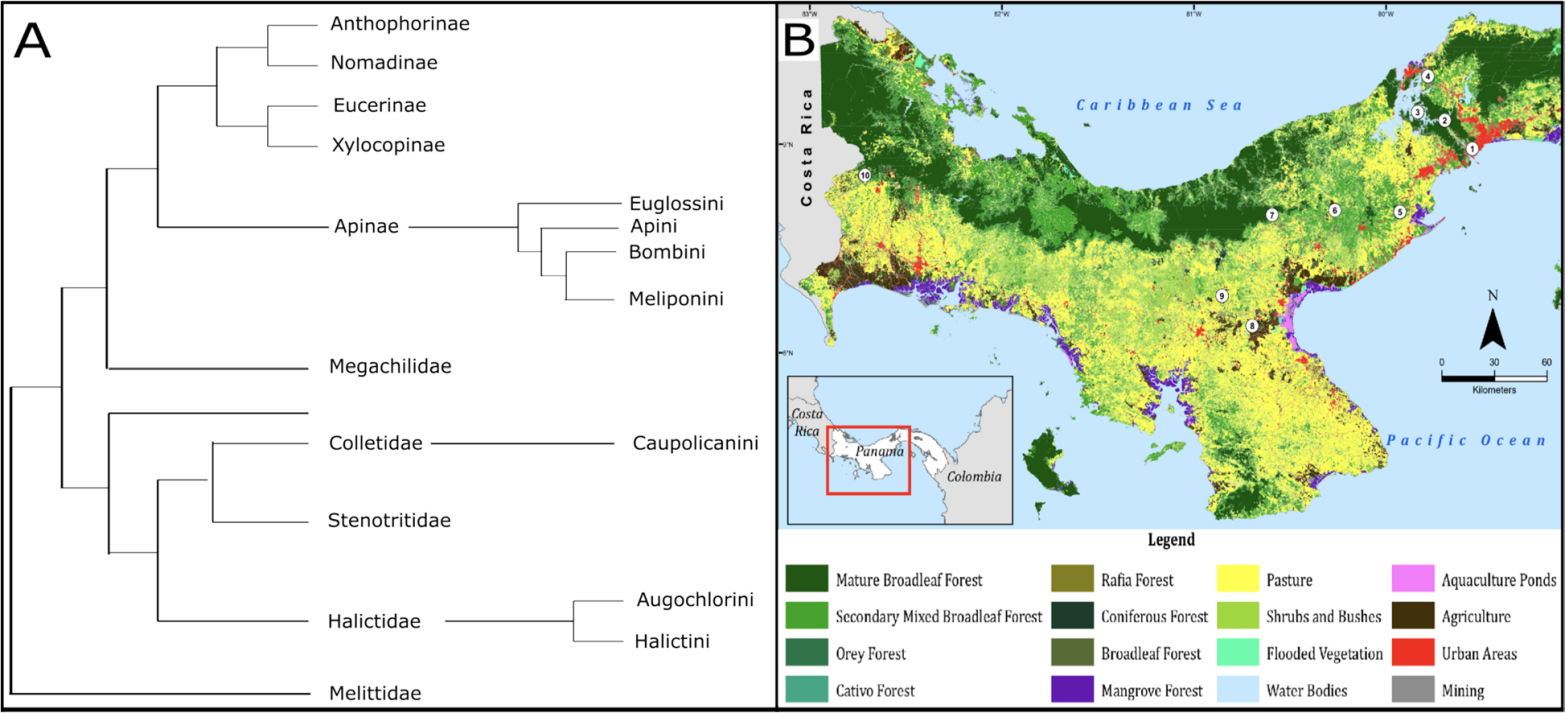
**A** Map of land cover in Panama displaying 10 locations sampled from 2018 to 2019. Site numbers (1–10) correspond to samples described in Supplemental metadata (tropical moist forest (1–3), tropical wet forest (4), premontane wet forest (5 and 7), tropical dry forest (6, 8, and 9), premontane rain forest (10), as described in [33]). **B** The phylogenetic relationships of bee tribes included in the study (following [34])

We submitted genetic material to generate amplicon sequence data for 912 bees, representing 51 species from 18 genera, seven tribes, and three families. A previously proposed phylogenetic relationship between bee tribes included in this study (Fig. 1A) demonstrates their extensive evolutionary range. Samples were collected from ten locations for 10 months, including wet and dry seasons. Protocol was established at *N*=6 for each bee species, per location and date, and was used for comparative study (Supplemental Table 1). Collecting, handling, and sampling methods are detailed in Supplemental Methods S2.1. Nets were sterilized to prevent possible spread of entomopathogens (see Supplemental Methods S2.1). Multiple species at a location were collected whenever possible; sites were sampled every 2 months; thus, natural flowering dynamics could be considered and evaluated [31, 32]. Forest types pertaining to bee sampling were premontane wet forest, premontane rain forest, tropical dry forest, tropical moist forest, tropical wet forest [33], mapped using ArcGIS Desktop 10 (2012, Ministerio de Medio Ambiente, Panama), and Political Boundaries [ESRI 2016] (Fig. 1B).

### DNA extraction, sequencing, and sample processing

Prior to DNA extraction, bee abdomens were homogenized using a bead for 2 min to ensure lysis buffers penetrated cells. DNA extraction employed a MoBio PowerSoil kit following EMP protocols (earthmicrobiome.org) [35]. DNA extracts were amplified from genomic DNA using PCR (515F/926R) primers: 5′-GTGCCAGCMGCCGCGGTAA-3′ and 5′-CCGYCAATTYMTTTRAGTTT [36]. All samples were submitted for 16S (V3-V4) amplicon sequencing with the Illumina HiSeq 2500 platform at The Hubbard Center for Genome Studies (HCGS), University of New Hampshire. Demultiplexing at HCGS employed bcml2fastq v2.20 and sequencing runs imported into Qiime2 v2021.4. All de-noising, trim parameters, taxonomy assignment, alignment, phylogenetic tree creation, and control sample processing were conducted in Qiime2 v2021.4; details are available in Supplemental Materials S2.2. After filtering, the resulting 16S feature table held 884 samples, 5,180,305 sequences with a median frequency of 2766 sequences per sample, and 9900 amplicon sequence variants (ASVs).

For bacterial diversity comparisons, we set a rarefaction depth of 899, which retained 595 samples, with 7359 unique ASVs (Supplemental Figure 1). Modeling analyses were duplicated using a rarefaction depth of 3500, which retained 393 samples, with 6900 unique ASVs, and the direction of the results is consistent. Additionally, we utilized compositionally aware analyses (Songbird [37]), as well as richness, and compositional assessments of CCGM that utilize that full dataset of 884 bees [38].

### Richness and composition analyses

*Alpha* and *beta* diversity were calculated using QIIME2 and its diversity plugin [39]. Faith’s phylogenetic diversity metric (Faith-pd; [40]) was selected because the ASV branch lengths between bacterial taxa were included. For higher resolution, we recorded “observed features” comparing rarefaction depths for bee species within tribes, and CCGM. Pielou’s evenness was calculated for microbial communities [41]. *Alpha* diversity and evenness were evaluated using Kruskal-Wallis tests and Wilcoxon rank-sum tests with Benjamini-Hochberg FDR (BH) corrections for significant differences [42, 43]. For brevity, results for Kruskal-Wallis are reported in the main text. Linear mixed effects models used to assess factors that may predict microbial richness (R packages (lme4, and glmulti [44, 45])) were visualized with tidyverse [46].

We conducted analyses of compositional differences using Songbird Qiime2 plugin, considering bee sociality, tribe, genus, species, forest type, and location [37]. Data parsing into testing and training datasets is explained in Supplemental Methods S2.3. When collected in three or more sites, bee species were tested for location effects. Microbial community structure was investigated with weighted UniFrac [47, 48]. Significant differences for *beta* diversity were calculated with permutational multivariate analysis of variance (PERMANOVA). To visualize differences in *beta* diversity metrics, we used principal coordinates analysis (PCoA).

### Differential abundance testing

We used Songbird to identify the bacterial taxa differing in abundance among tribes, genera, species, locations, forest types, and social categories (tables in Supplemental Materials—Songbird Tables). Seasonal differences within species collected at the same location were analyzed using the DESeq2 package in Phyloseq R (Rversion2021) (methods in [49]). We visualized taxa with tidyverse [46], provided in the Supplemental Materials (Seasonal Taxa).

### Proportional abundance tables

Feature tables were imported into R, grouped by bee tribe, and summarized by genus and species (where N>3 per species). The 10 most abundant bacterial families (here called “dominant” bacteria) from Euglossini, Meliponini, and “other” bees were determined. We combined them to visualize the composition of the 16 shared bacterial families and to group the rest of the bacterial diversity within the category “Other”.

### Core microbial regression analysis

Using 5% increments from 50 to 100%, we calculated the number of microbial taxa that were present for each bee tribe and sociality category and plotted the values. We considered the shared microbes present at each increment to be the “core” microbes at that depth.

### Lowland tropical forest bee sub-analyses

To remove location effects and other sources of variation, we subsampled our dataset to include only the lowland tropical forest sites. They provided the most in-depth sampling of Euglossini (facultatively social at these sites) and Meliponini (highly eusocial) through time. We (1) modeled temporal effects on microbial richness, (2) computed the monthly richness and evenness of each tribe using Faith-pd in Qiime2, (3) calculated the core microbial regression (described above), and (4) compared the number of ASVs by tribe (Euglossini and Meliponini), for the month with the most samples (May; Meliponini *N*=26 samples, and Euglossini *N*=32 samples) and visualized the overlap of microbial taxa using a Venn Diagram (Venny 2.0).

### Seasonal comparison of bee richness

We compared bees of the same species at the same locations during wet and dry seasons. The mean effect sizes (Hedge’s G—bias-corrected standardized mean differences; Hedges 1981) were compared across 10 species for alpha diversity (ASV richness, Faith-pd, Shannon, and evenness), which were visualized using ggplot 2 and reshape [50].

### Bee morphometric comparisons

For each bee species, tongue length, body length, and color data were recorded and given rank scores: tongue length (1–4), body length (1–5), and body color (1–3). Methods are provided in the Supplemental materials and values per species are provided in Supplemental Table 1. We further examined the contribution of morphology in Euglossini and Meliponini independently, and these results are available in the Supplemental Rmarkdown.

### Richness and composition of CCGM

We calculated the percentage of each CCGM (*Snodgrassella*, *Gilliamella*, *Lactobacillus*, and *Bifidobacterium*) for each tribe and social category. We displayed the relative abundance of CCGM on a log scale, to incorporate the exponential nature of PCR. Additionally, we calculated the number of CCGM variants for each bee species. The *Lactobacillus* clades Firm-4 and Firm-5 have been highlighted in other studies [23, 26, 51], but we chose not to eliminate diverse *Lactobacillus* taxa because specific relationships with bees are generally unknown [52]. Data filtering for CCGM is further described in *S3.7.*

## Results

### Comparative bee microbiota, internal versus external

We found no significant differences in bacteria composition of dissected bee guts versus whole abdomens for the 7 genera and 16 species tested with sufficient data (Supplemental Table 2).

### Predictors of bee microbial richness

**Fig. 2:**
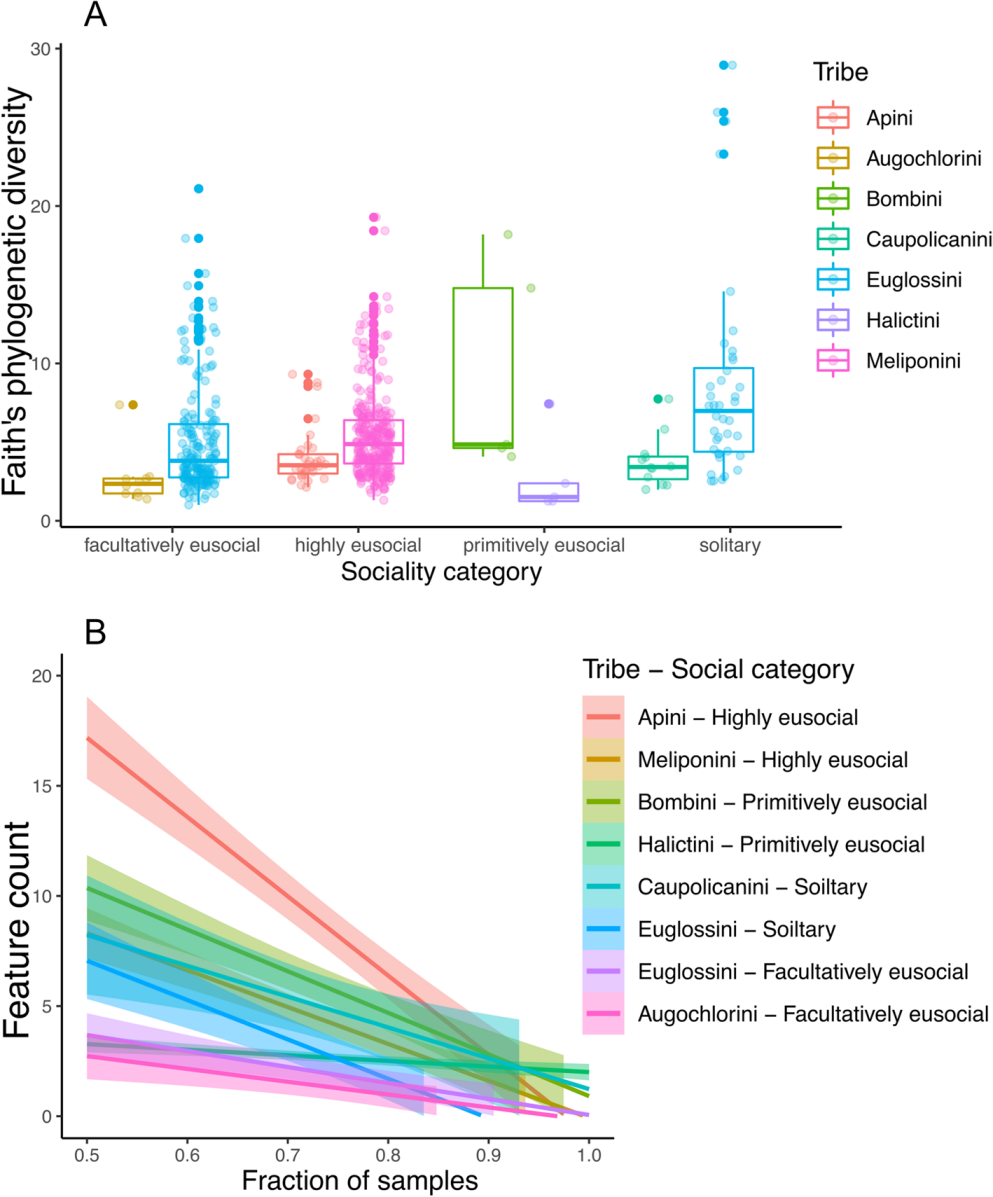
**A** Faith’s phylogenetic diversity of bee samples arranged by social category and colored by bee tribe. The overall statistical difference between social categories is *χ*^2^=8.54, df = 3, *p* = 0.036. **B** The core feature counts by the fraction of samples. Linear regressions show 95% confidence intervals (above zero) colored by tribe and social category

We identified statistically significant differences across facultatively social, highly eusocial, primitively eusocial, and solitary bees (Fig. 2A — *χ*^2^=8.54, df = 3, *p* = 0.036). Our best model held sociality as a fixed effect, and species within location as random factors. Highly eusocial and primitively eusocial bees were not distinct from facultatively social bees (Tukey’s: *z* = 1.6; *p* = 0.34, and *z* = 0.32; *p*= 0.98) respectively, but solitary and facultative social differed from each other (Tukey’s: *z* = 2.8, *p*= 0.022). Within euglossines, facultatively eusocial bees had lower microbial richness compared with solitary, and they differed significantly (Kruskal-Wallis; *H* = 15.78, *p* ≤0.001), and within the solitary euglossine bees, the more seasonal genus *Eufriesea* had substantially increased microbial diversity over the parasitic genus *Exaerete*. The parasitic species, *Exaerete smaragdina*, had significantly higher diversity than its host *Eulaema nigrita* (Kruskal-Wallis; *H* = 6.866, *p* = 0.009), but this association was not significant for *Exaerete frontalis* and its host *Eulaema meriana* (Supplemental Materials—Host-Parasite Comparison).

The number of shared bacterial taxa found within tribe and social category was calculated across the percentage of samples collected within each group, its core, using 5% increments. Highly eusocial bees were more likely to share bacterial taxa with each other and maintained higher levels of core members across core thresholds than primitively eusocial, solitary, and facultatively eusocial bees (Fig. 2B). Euglossini, Meliponini, and those grouped as “other” bee species significantly varied in their bacterial diversity Supplemental Figure 2.

### Bee microbial predictors and community structure

We assessed microbial composition using Songbird for both individual variables and combined (additive) models. The variables tribe, genus, species, location, forest type, sociality, and seasonality all accounted for additional variation, compared to the null model. The designation of a species was the best predictor of microbial composition for all bees (38.2% variation explained), Euglossini (15.3%), and Meliponini (39.8%) (Supplemental Table 3). The interaction between sociality and species explained more variation than their summation independently, and this was particularly true for euglossine bees. Results of PERMANOVAs were also highly significant (tribe, *F* = 9.497, *p* < 0.001; genus, *F* = 9.038, *p* < 0.001; species, *F* = 6.112, *p* < 0.001; social category, *F* = 11.165, *p* < 0.001). Monthly comparisons for each factor were all significant and presented in Supplemental Table 4. Visualizations of both tribe and social category are provided in Supplemental Figure 3. The microbial composition of host-parasite pairs, *Exaerete frontalis and Eulaema meriana*, as well as *Exaerete smaragdina and Eulaema nigrita*, differed moderately (*F* = 1.85, *p* = 0.081; and *F* = 2.55, *p* = 0.022, respectively).

**Fig. 3:**
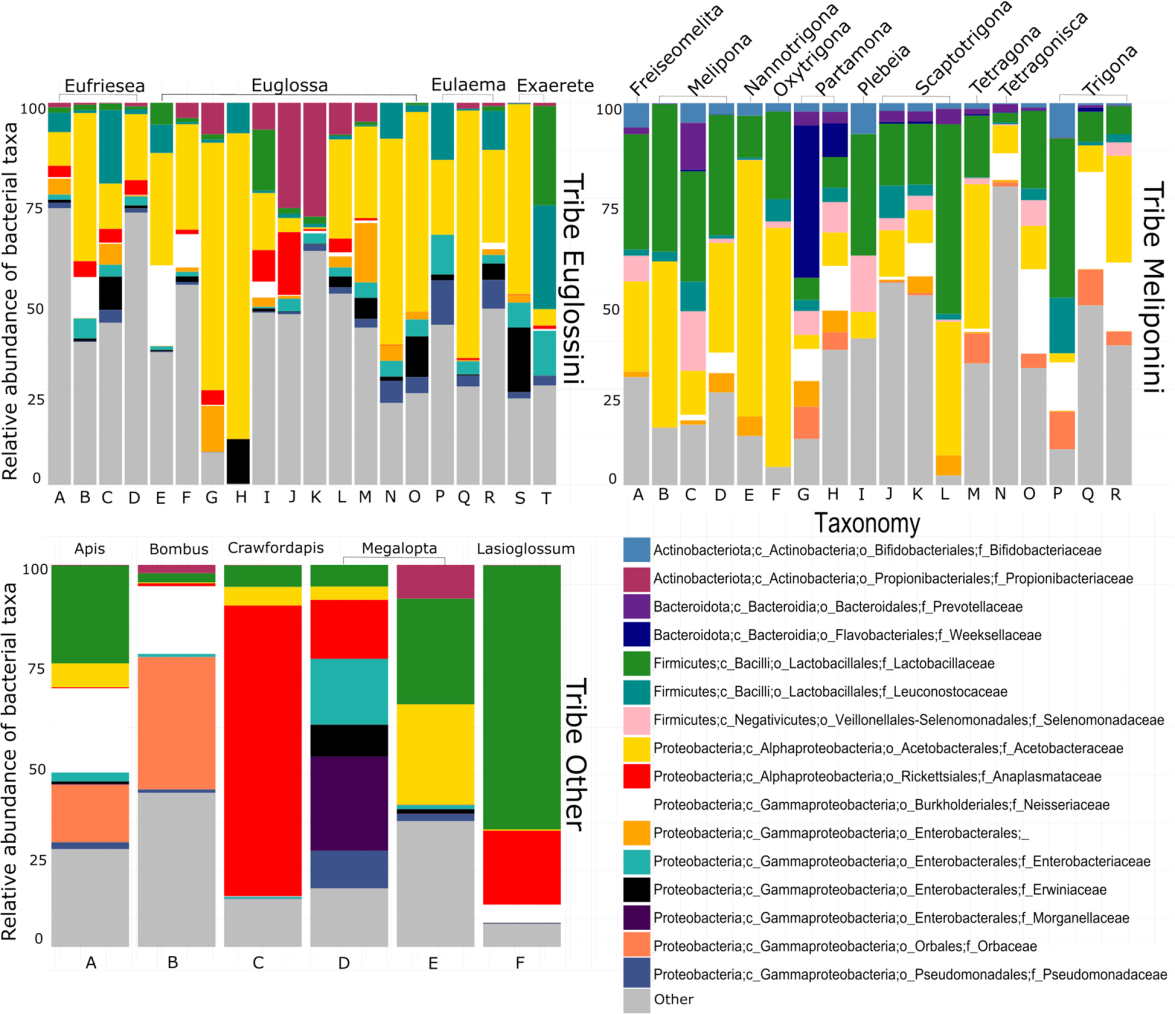
The proportional abundance of ASVs in the microbiome of diverse bees collected across locations in Panama. Proportional abundances are arranged in three groups, tribe Euglossini (*N*=20), tribe Meliponini (*N*=18), and tribe Other (*N*=6). Taxonomy is arranged in descending relative abundance within the respective panels. Panel 1 Tribe Euglossini is arranged as follows: *(A) Eufriesea pulchra, (B) Eufriesea rufocauda, (C) Eufriesea anisochlora, (D) Eufriesea chrysopyga, (E) Eulaema meriana, (F) Eulaema nigrita, (G) Eulaema bombiformis, (H) Euglossa cognata, (I) Euglossa championi, (J) Euglossa sapphirina, (K) Euglossa tridentata, (L) Euglossa asarophora, (M) Euglossa bursigera, (N) Euglossa imperialis, (O) Euglossa mixta, (P) Euglossa crassipunctata, (Q) Euglossa deceptrix, (R) Euglossa dodsoni, (S) Exaerete frontalis, (T) Exaerete smaragdina.* Panel 2 Tribe Meliponini is arranged as follows: *(A) Frieseomelitta nigra, (B) Melipona phenax, (C) Melipona triplaridis, (D) Melipona panamica, (E) Nannotrigona perilampoides, (F) Oxytrigona mellicolor, (G) Partamona peckolti, (H) Partamona musarum, (I) Plebeia frontalis, (J) Scaptotrigona panamensis, (K) Scaptotrigona barrocoloradensis, (L) Scaptotrigona luteipennis, (M) Tetragona ziegleri, (N) Tetragonisca angustula, (O) Trigona fulviventris, (P) Trigona almathea, (Q) Trigona ferricauda, (R) Trigona corvina.* Panel 3 Tribe Other is arranged as follows: (A) *Apis mellifera*, (B) *Bombus volucelloides*, (C) *Crawfordapis lutcuosa*, (D) *Megalopta amoena*, (E) *Megalopta genalis*, (F) *Lasioglossum umbripenne*

We compared the 16 dominant bacterial families across bee tribes. Acetobacteraceae, Lactobacilliaceae, and Enterobacteriaceae were proportionally abundant in Euglossini, Meliponini, and in “other” (Fig. 3). Bifidobactereacae, Prevotellaceae, Leuconostocacae, and Selenomonadaceae were foremost among stingless bees, but were uncommon in other bees, except in *Megalopta amoena* and *Exaerete smaragdina*. Propionibacteriaceae were dominant in euglossine bees, particularly the genus *Euglossa*, but also found in *Bombus volucelloides* and *Megalopta genalis*. Pseudomonadaceae and Erwiniaceae occurred in Euglossini and the composite group “other,” but not in stingless bees, except Pseudomonadaceae in *Partamona*. Neisseeriaceae (including *Snodgrassella*) was found in 12/18 stingless bees, 8/20 euglossine bees, and 3/6 bees in “other,” to varying degrees. Orbaceae (including *Gilliamella*) had a similar distribution. Anaplamataceae (including *Wolbachia*, largely absent in our study and other microbial work in bees) was notable in bees of relatively low microbial diversity, *Crawfordapis luctuosa* and *Lasioglossum umbripenne*, but occurred in samples of other taxa as well (e.g., some Euglossini).

### Lowland tropical forest sub-analysis

We found Meliponini maintained more diverse and more even microbial communities compared to Euglossini (Supplemental Figure 4A, B). Their core microbes were also more diverse (fourfold) than those of euglossine bees (Supplemental Figures 4C). More specifically, at the start of the wet season in May, euglossine and meliponine bees shared 245 ASVs. The percentage of overlap for euglossine bees was 43.6% (245/562), and the percentage of overlap for meliponine bees was 16.5% (245/1488), see (Supplemental Figures 4D).

**Fig. 4:**
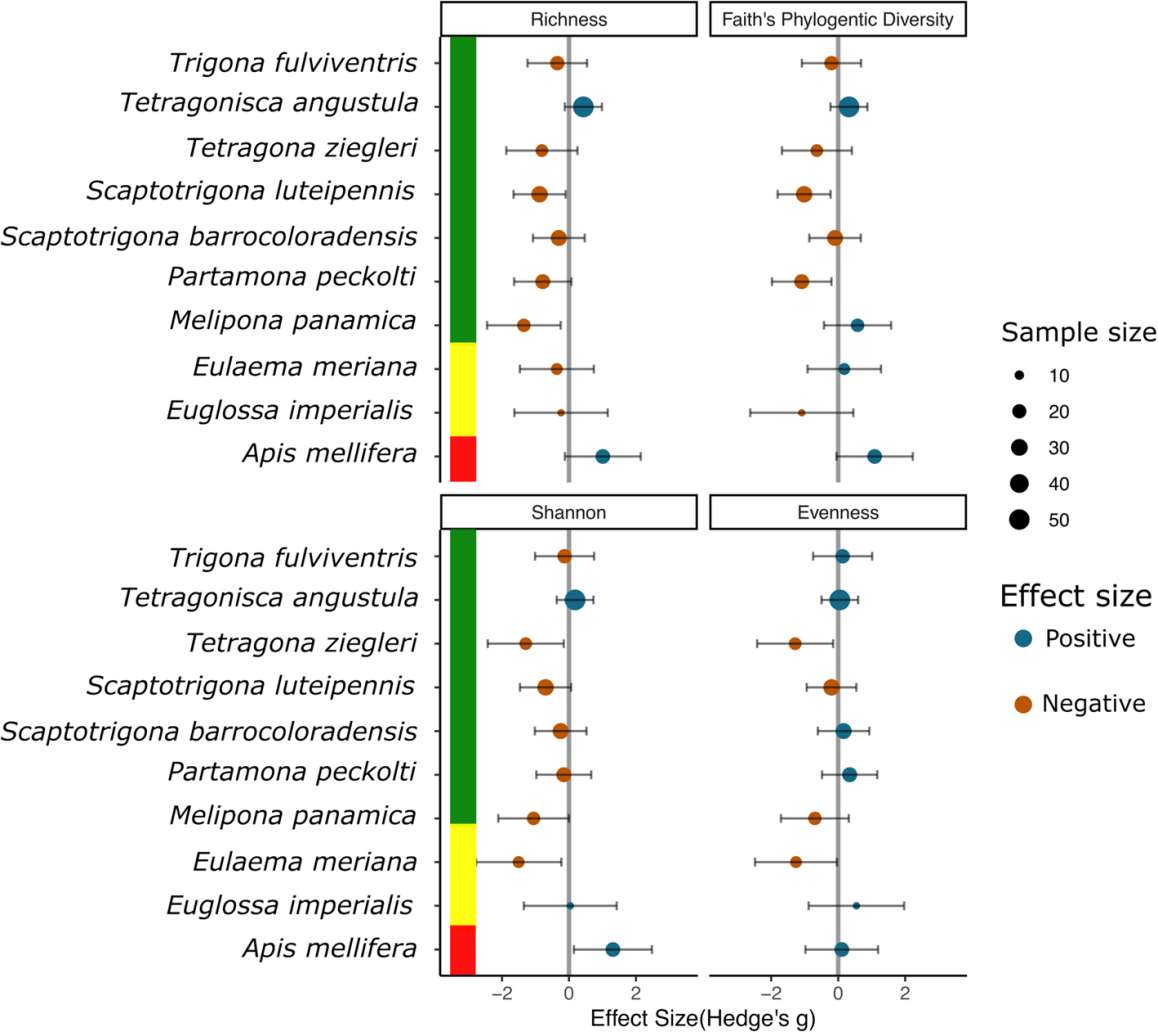
A comparison of the microbiome of bee species sampled from the same location, during the dry season and wet season (excluding transitional months). The magnitude and direction of effect sizes vary across the bee species studied. Four panels show the mean effect sizes (Hedge’s G—bias-corrected standardized mean difference) across 10 studied species for alpha diversity (ASV Richness, Faith’s Phylogenetic Diversity, Shannon, and Evenness). Negative (red) values indicate a reduction in the dry season, while positive values (blue) indicate an increase in the dry season. Point size is scaled by sample size. Overall standard mean difference (SMD) and random effect model *p*-values are provided for each metric. Vertical color bars indicate bee tribes (Tribe Meliponini (*N*=7; green), Tribe Euglossini (*N*=2; yellow), and Apini (*N*=1; black)

### Seasonality as an explanatory variable of bee microbial diversity

We calculated effect sizes (Hedges g) and compared the microbiome of 10 species collected at the same locations across wet and dry seasons, excluding transition months. For most bees, microbial diversity was significantly lower in dry season compared to the wet season, and this was true for ASV Richness, Shannon diversity, and Faith’s phylogenetic diversity (SMD −0.35, *p* = 0.02, SMD −0.32, *p* = 0.02, and SMD −0.18, *p* = 0.01, respectively). The effect size for Pielou evenness was not significant (SMD −0.11, *p* = 0.21), but trended toward reduced evenness in the dry season versus wet season (Fig. 4). Furthermore, we investigated the bacterial taxa (ASVs) that were seasonally constrained. We used DESeq2 and found wide variation in responses depending on host and microbe taxa. A summary of bacterial taxa that are differentially abundant across season, many of which have known or supposed functions, can be found in *S3.5* and Supplemental document DESeq2, by season.

### Bee morphometrics and microbial diversity

**Fig. 5:**
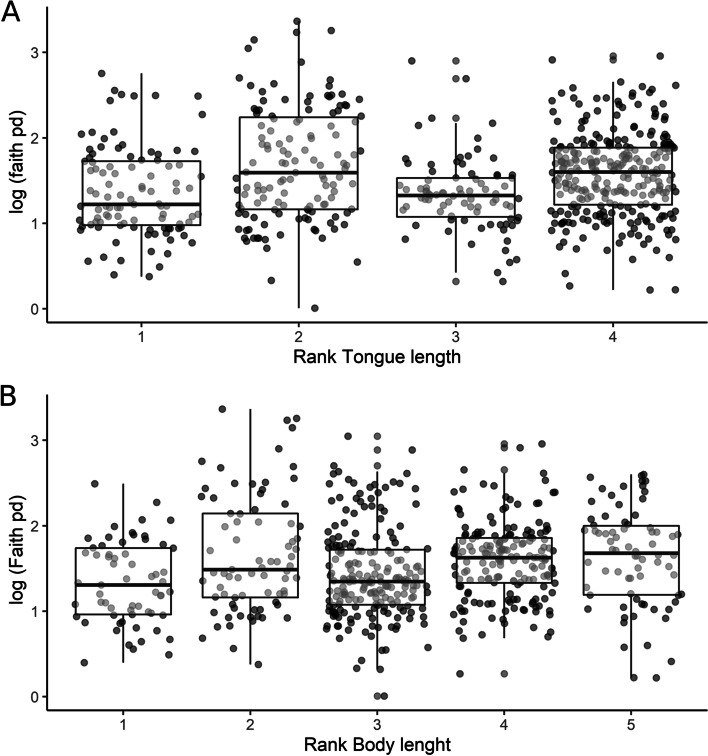
Bee morphometric data for all bee samples relative to microbiome diversity. Tongue length is the trait that best predicts microbial richness among bees. **A** Faith’s phylogenetic diversity arranged by rank tongue length (1 is the longest and 4 is the shortest), showing significant differences across subcategories. **B** Faith’s phylogenetic diversity of bee species arranged by body length (1 is the longest and 5 is the shortest), showing significant differences across subcategories

Model selection determined that tongue length and secondarily, body length, although correlated, differed significantly across rank values for all bees (tongue length; *χ*^2^ = 12.39, df = 3, *p* = 0.0062: Body length; *χ*^2^ = 15.92, df = 4, *p* = 0.0031). Both explanatory variables were retained in our model of morphology and microbial richness. Our most informative model held rank tongue length and body length as fixed effects, and location, collection season, and genus as random effects. Bees with longer tongues had substantially reduced microbial richness (Fig. 5). We also observed body length was inversely proportional to microbial richness across all bees. To better understand how body morphometric traits correlate with bee microbiomes, we analyzed morphometric traits within Euglossini and Meliponini alone. We found tongue length was a strong predictor of microbial richness in euglossine bees (*χ*^2^= 6.63, df=1, *p* = 0.01; Supplemental Figure 5). This was most evident in *Eufriesea*, which has the largest richness differential of any euglossine genus (ranging from an average of 19–125 unique ASVs per bee (*Eufriesea pulchra* and *Euf. anisochlora*, respectively)* —* and represents maximum differences in tongue length. The relationship between morphometric traits and microbial richness in meliponine bees was minimal, but body length was inversely proportional to microbial richness in both Meliponini and Euglossini (Supplemental Figure 5, Supplemental Figure 6). The results are in Supplemental materials (S3.6).

**Fig. 6:**
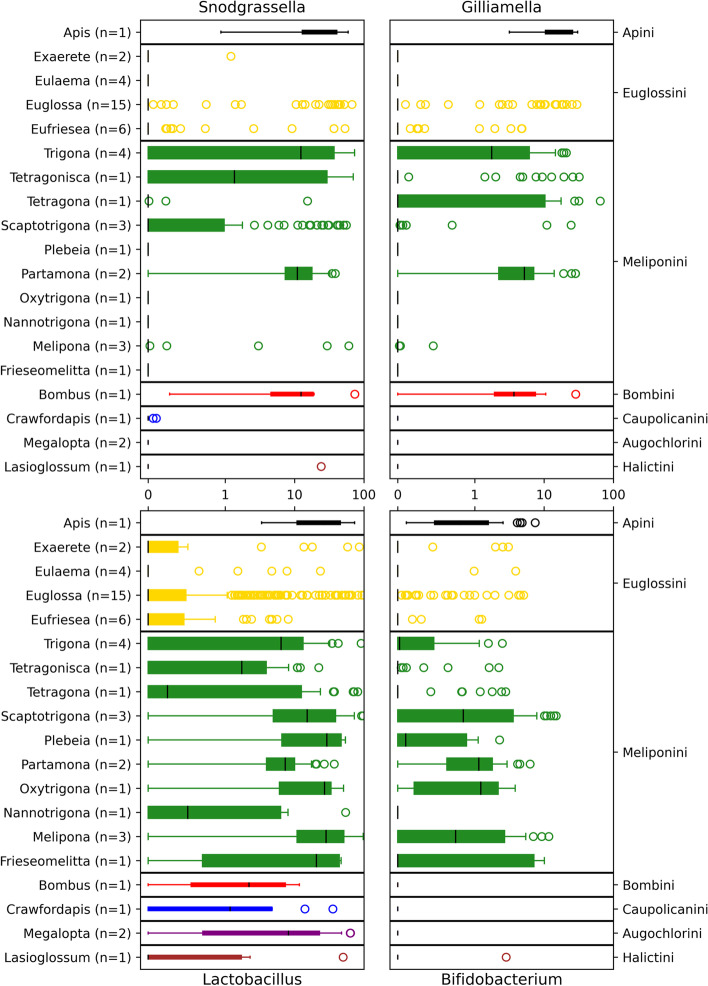
Plots the CCGM and their distributions (percent relative abundance per sample) for all bee genera. The number of species included in each bee genus is reported. Boxplots are color-coded by bee tribes

### Alpha diversity of CCGM microbiota

We found sequence variants of *Snodgrassella* (*N*=130 variants), *Gilliamella* (*N*=56), *Lactobacillus* (*N*=407), and *Bifidobacterium* (*N*= 64) among some species of all bee tribes in the dataset. Apini, Meliponini, and Euglossini had all four CCGM taxa; our single species of Bombini lacked *Bifidobacterium*, and our single species of eusocial Halictini lacked *Gilliamella*, while only *Lactobacillus* occurred in Augochlorini and Caupolicanini. We summarize the richness, composition, and percentage of the CCGM for bee tribes and social categories in Supplemental Figure 7. The percentage of the CCGM was highest in Apini—highly eusocial at 66%, and lowest in Euglossini—nonsocial or eusocial (generation overlap) at 4%. Although high variation occurred in Meliponini, CCGM were less abundant than in *Apis mellifera*, and the greatest number of outliers was found among Euglossini.

We assessed the percentage per sample of each CCGM at the bee genus (Fig. 6) and species levels (Supplemental Figure 8). We found that *Snodgrassella* was consistently present in 4 of 10 (40%) of meliponine genera (Scaptotrigona, *Trigona*, *Tetragonisca*, and *Partamona)* and 8 of 18 (44%) meliponine species. *Partamona* often nests in aggregations, and both *Trigona* and *Partamona* extensively use pollen exine and/or feces to make their nest envelopes [53]. *Snodgrassella* was also present in *Bombus* and *Apis*. *Gilliamella* was consistently present in 3 of 10 (30%) meliponine genera (*Trigona*, *Partamona* and *Tetragona) and* 7 of 18 (39%) meliponine species, and in *Bombus* and *Apis. Lactobacillus* were universally present across all bee tribes and genera, except for *Eulaema* (found with the highest occurrence in *Euglossa cybelia* and *Exaerate smaragdina*, *Trigona corvina*, *Scaptotrigona barrocoloradensis*, *S. panamica*, *Melipona triplaridis*, and *M. phenax*). In general, euglossine genera had low probabilistic occurrences of all CCGM, except for some species (*Exaerate smaragdina*, *Euglossa asarophora*, *Eug. bursigera*, *Eug. crassipunctata*, *Eug. cybelia*, *Eufriesea anisochlora*, *Euf. chrysopyga*, *Euf. pulchra*, and *Euf. rufocauda*). *Bifidobacterium* was present in 7 of 10 meliponine genera, and *Apis*, but not in *Bombus*. Additional bee genera had multiple occurrences of CCGM (captured by the upper quartile) but retained a mean percent sample occurrence close to zero.

The richness of CCGM by bee species is shown in Supplemental Figure 9. Thought to occur in the ilium of bee species, 130 variants of *Snodgrassella* were found among 26/51 bee species. The highest level of variation was found in *Tetragonisca angustula* (*N*=10, with an average of 4 variants per individual), whereas individuals of several species of *Euglossa*, *Eufriesea,* and *Crawfordapis* had only one detected variant. Notable exceptions include several individuals of *Euf. pulchra*, *Eug. asarophora*, *Eug. bursigera*, and *Eug. crassipunctata*, which were found with two to three variants of *Snodgrassella*. Variants of *Gilliamella*, found in 23 of 51 bee species, were less diverse (*N*=56), and the highest level of variation was found in *Tetragona ziegleri* (*N* = 5, with an average of 3 variants per individual). *Partamona peckolti* and *Apis melifera* had up to 4 variants. The same euglossine species that associated with *Snodgrassella* also were found with *Gilliamella.*

Thought to occur in the rectum, 407 variants of *Lactobacillus* occurred in 44 of 51 bee species. The highest level of sequence variation was found in *Lasioglossum umbripenne* (*N*=23, with an average of 13 variants per individual). Other bee species, such as *Melipona triplaridis*, *Scaptotrigona luteipennis*, *Scaptotrigona pectoralis panamensis*, *Tetragona ziegleri*, and *Trigona ferricauda*, each had considerable variant diversity (*N* > 15). Lower diversity of *Lactobacillus* (*N* < 5) was typical for euglossine bees. Lastly, 64 variants of *Bifidobacterium* were found in 31 of 51 bee species, and the highest level of sequence variation was found in *Partamona peckolti* (3^rd^ quartile *N* = 7, with an average of 4 variants per individual). *Bifidobacterium* was typically absent in euglossine bees, except in *Exaerate smaragdina*, *Eug. crassipunctata*, and *Eug. asarophora*, which along with several others previously mentioned, were associated with most CCGM.

### Differential abundance analysis of CCGM in bee species

We explored bacterial taxa including CCGM that contributed to differences between tribes, genera, species, locations, forest types, seasons, and social categories. Here, we report on the CCGM that are found to contribute to differences in bee species — the best predictor of microbial composition (Supplemental Table 2). All contributing features can be found in the supplemental materials (Songbird Tables). By filtering CCGM by bee species, we detected 4 variants of *Gilliamella*, 8 variants of *Snodgrassella*, 20 variants of *Lactobacillus*, and 8 variants of *Bifidobacterium* that contributed to compositional differences among species, and we see evidence of strong association of bee species of varying life history strategies with specific CCGM (Supplemental Songbird differential features CCGM).

Certain bees also displayed strong positive associations (scores >3) with multiple variants within the same CCGM genera. *Euglossa crassipunctata*, for example, had multiple strong positive associations with all CCGM except *Bifidobacterium. Tetragonisca angustula* and *Eulaema meriana* had multiple strong positive associations with *Snodgrassella. Melipona phenax*, *M. panamica*, *M. triplaridis*, and *Tetragona ziegleri* had multiple strong positive associations with *Snodgrassella and Lactobacillus. Partamona musarum* and *P. peckolti* had multiple strong positive associations with *Gilliamella* and *Bifidobacterium. Lasioglossum umbripenne*, *Scaptotrigona barrocoloradensis*, *S. luteipennis*, and *S. panamensis* had multiple strong positive associations with *Lactobacillus.* Lastly, *Trigona fulviventris* had multiple strong associations with *Snodgrassella and Bifidobacterium.* Thus, affinities for multiple variants of the same CCGM and affinities of single and pared CCGM associations were found across diverse groups of bees.

## Discussion

The bee microbiome’s crucial importance to bee fitness is suggested in studies of a few species [8]. The general biotic and abiotic factors that determine a bee’s microbiome are not well documented. In our analyses, we explored patterns of microbial diversity among tropical bees of differing life histories and reproductive strategies, morphological characteristics, and seasonality. A robust CCGM was present in several but not most eusocial bees with vertical microbial transmission, which may have been lost in some. These CCGM presented more variability in eusocial bees with annual colony cycles compared to perennial colonies. Intriguingly, CCGM were also found in some solitary species.

The overall differences in microbial diversity found for bee species, as well as across seasons may provide some insight into the microbiome dynamics within flowers. Flowers are hypothesized to function as dispersal hubs for microbes, and both social and solitary bees may acquire microbes as they also acquire resources [26]. Our data suggest a negative correlation between bee body size, tongue length, and microbial richness. Hypotheses underpinning morphological differences and their proposed influence on host-microbial diversity, stemming from restrictive flower visitation, is most clearly represented in euglossine bees and discussed below. Our data suggest also there may be more microbial richness on flowers in the wet season compared with the dry season, and conversely more distantly related microbial taxa on flowers in the dry season. However, enhanced diversity in the wet seasons could be the result of diverse flower visitation, as many plants flower early in the rainy season, and diverse pollen sources are relatively more abundant then [54, 55]. Alternatively, bees that are not seasonal may become more specialized on heavily flowering species during the dry season [56], thereby reducing their exposure to transient microbes at floral hubs.

Floral-associated acid-producing microbes that may play roles in pollen preservation and nutrient liberation which can influence bee larval development were consistently present [26, 57]. Bacteria in the family Acetobacteraceae, which included acetic acid-producing bacteria [58] (Acetic acid ~4.8 pKa) in the genera *Asaia*, *Bombella*, *Commensalibacter*, *Endobacter*, *Gluconacetobacter*, *Gluconobacter*, were detected across diverse bees but were notably absent in Bombini. In addition, lactic acid bacteria [59] (Lactic acid ~3.86 pKa) in the genera *Lactobacillus*, *Carnobacterium*, *Lactococcus*, *Streptococcus*, *Enterococcus*, *Leuconostoc*, *Pediococcus*, and *Weissella* were generally present across diverse bees, while *Vagococcus*, *Oenococcus*, *Tetragonococcus*, and *Aerococcus* were only found in trace amounts. *Pediococcus*, however, had a dominant association with Halictini, which was not observed in any other bee tribe.

Euglossines exhibited high levels of microbial variability, which may occur due to the variability in the biology of the bees themselves, specifically, their seasonality (univoltine vs. multivoltine), their tongue length that regulates nectar gathering behaviors, and their degrees of sociality and parasitism. Despite high variability of euglossine gut microbiomes, the predictive power of tongue length for euglossine microbial richness provides a clear explanation, resulting from a natural experiment on a measurable trait. Euglossine bees have more varied tongue lengths than the other bee tribes [24]. Shorter-tongued euglossine bees may have higher exposure to microbes from less restrictive flowers that support more visitors including bees, wasps, butterflies, moths, beetles, etc. [60]. In contrast, long tongues (sometimes exceeding the length of their body) are specialized to forage from deep flowers that are often designed to exclude other visitors. As a result, they may be less exposed to transient microbes at more widely utilized floral hubs. Our dataset fits this hypothesis; *Euglossa asarophora*, *Eg. imperialis*, *Eulaema bombiformis*, *El. cingulata*, *El. meriana*, *El. nigrita*, and *Exaerete frontalis* have the longest tongues, and each had a lower-than-average microbial richness. *Eufriesea anisochlora*, which had the highest microbial richness of any bee species, also had the shortest tongue among the euglossine bees*.* This observation likely explains why the microbiome of *Eufriesea anisochlora* had significant overlap with CCGM’s of many eusocial taxa. The variability in microbial diversity and composition of *Eufriesea* suggests they exemplify seasonal and floral-mediated microbial dynamics from frequent inoculations of microbes at their food resource flowers (D. W. Roubik and J. E. Moreno, unpublished manuscript). *Eufriesea*, specifically the two species with the highest or lowest bacterial diversity, also had the shortest or longest flight seasons [24], respectively, and therefore may logically be exposed to differing bacterial communities. From a theoretical standpoint, the fittest microbes would eliminate competitors and may alter the health of the host [61]. However, selection on microbes and thereby their hosts may not be as strong for animals with very short lives — a concept well established in the disease ecology literature [62, 63].

That we sampled male euglossines for pragmatic reasons (bait attraction for males only) while other species were sampled from females does not seem pivotal here, and there are some biological reasons why male euglossine bees may serve as a proxy for females of the species, particularly at the scale that we are investigating. Principally, males are relatively long-lived, and males have been shown to acquire pollen and presumably microbes at flowers from previous visits by females ([64], D. W. Roubik and J. E. Moreno, unpublished manuscript). Indeed, more detailed comparison of both genders in orchid bees would serve to test the hypothesis that their acquisition of microbes at flowers does not differ significantly. Further discussion of the potential for differences in the microbiome of sexes of bees can be found in Supplementary Discussion *S4.2.*

The highly eusocial stingless bees were more stable in microbial associations than orchid bees, typically dominated by Acetobacteraceae, Lactobacillaceae (containing *Lactobacillus*), Neisseriaceae (containing *Snodgrassella*), Orbaceae (containing *Gilliamella*), Bifidobacteriaceae (containing *Bifidobacterium*), and Selenomonadaceae. The more even microbial associations may have occurred due to the stabilizing forces of long-term perennial colonies with eusociality that enable vertical transmission and the availability of stored foods within a hive. The stingless bees have storage areas where microbes process stored pollen, honey, and even carrion for the few species that are obligate necrophages [23]. Bees that also use meat and feces (e.g., *Trigona*, *Partamona*) have particularly strong associations with bacteria of the genera *Gilliamella* and *Snodgrasella*, which are known to help detoxify compounds [53]. Such differing life history strategies highlight the significant knowledge gaps that exist in our understanding of how bees manage their microbial associations in relation to resource utilization. Further still, other insects such as flies that visit flowers and strongly associate *Gilliamella* [65] may refine our understating of the metabolic functions that certain microbes perform for hosts and the degree of host specificity and horizontal transfer of CCGM taxa that can occur at flowers.

## Conclusions

The notion of a core microbiome for corbiculate bees [23] is based on limited spatial and temporal sampling, often including perennial colonies relocated by humans. In this study, we found key microbial taxa present in several, but not all eusocial corbiculate bees, also reported elsewhere [23]. However, many of these bacterial taxa are also present in bees with different behaviors, raising questions about the generality of core bacterial genera — regardless of sociality. For example, only 3 of 10 stingless bees had simultaneously proportionally abundant and consistent associations with *Gilliamella* and *Snodgrassela*, contrary to the hypothesis that these taxa are central microbes to all or most social corbiculates. Furthermore, stingless bee colonies that have been moved can show dramatic shifts in their microbial associations, such as the loss of CCGM, which were not restored upon their relocation at the original site [30]. Therefore, eusociality may have a limited explanatory power concerning bee microbial associations. Other attributes, such as life-history strategies, as well as horizontal transmission of beneficial microbes at flowers, may prove more informative and predictive of bee microbiomes. Our data suggests that highly eusocial bee nests, and likely the flowers they visit, may act as hubs for other bees that frequently acquire and lose CCGM. Indeed, many bee species may rely on microbes provided through horizontal transmission by neighboring perennial bee colonies, and community-wide transmission pathways should be explored. Lastly, for most microbes, we lack functional understanding of their roles in bee biology. Yet undeniably, many bee-microbial associations appear to be with acetic and/or lactic acid fermenters. Thus, understanding microbial associations of bees requires a community-level approach, that also investigates bee microbiomes, and their functional roles, at scales commensurate with bee species life cycles, even across seasons or years.

## Supplementary Information

**Additional file 1.** Supplemental Materials. Supplemental Figures. Supplemental Tables

## Data Availability

The raw data generated and additional supplemental data files, figures, and tables are available on Dryad repository (Link available Upon acceptance).
