# Supplementary material for: Neotropical bee microbiomes point to a fragmented social core and strong species-level effects
Source: Microbiome. 2023 Jul 14;11:150. doi: 10.1186/s40168-023-01593-z (PMC10347802; doi:10.1186/s40168-023-01593-z)
Supplement: Supplementary file 2 — Additional file 1. Supplemental Materials. Supplemental Figures. Supplemental Tables [file 40168_2023_1593_MOESM1_ESM.docx]

**Supplemental Materials**

**S1. Supplemental introduction**

*S1.1 Bee Natural History & Flora of Panama*

Bees form a monophyletic group derived from predatory sphecoid wasps and comprise seven families [1]. Here we present principal natural history details that likely relate to microbial diversity. With the exceptions of obligately necrophagous species and bees that feed on honeydew from sap feeding insects, bees depend primarily on flowering plants that provide resources (nectar, pollen, floral oils) for larval development and adult nourishment. These resources are gathered and cached within individual brood cells and also stored in eusocial bee nests within waxen or wax and resin containers. Depending on species, the viscosity of brood provisions ranges from a stiff dough-like mass, the consistency of yogurt, or a soupy liquid. Nest cells are lined with Dufour’s gland chemicals in many taxa, which may be added to the provision mass [2]. Building materials range from mud and compacted soil, to wood, resins and wax. Life cycles range from uni- to multi-voltine annual reproduction, to the perennial colonies of highly eusocial species that have reproductive division of labor. Larvae of some species are progressively fed, while in most species, cells are mass provisioned with all the food a larva will receive. Among bees, some species are pollen generalists (polylectic) and others are specialists (oligolectic) [3], but all species in our study are pollen generalists, insofar as data are available. Our samples come from adult bees in Panama, where there are two broad flowering peaks in the year, one in the dry season and another in the early wet season [4, 5]. The wet season is associated with release of many microbes from their dormant states [6].

*S1.2 Bee taxa included in this study*

APIDAE

The Apidae are a diverse family of long-tongued bees, with more tribes than any other family [1]. The behavior of female apid bees is equally diverse, ranging from solitary female nests to highly eusocial caste-based societies, and many genera or subgenera are parasitic on the stored food or brood of other related bee genera [2, 7]. Diet preferences (pollen specialization) vary among and within genera. Females in the tribes Apini, Meliponini, Euglossini and Bombini have modified hind legs with corbiculae for transporting materials to the nest and together are the corbiculate apids; nearly all are foraging generalists.

Apini: The Apini contain about a dozen species of stinging honey bees in the genus *Apis [1].* *Apis* is primarily tropical and historically was restricted to the Old World. Today *Apis mellifera* is distributed world-wide due to human activity. These highly eusocial bees have large colonies ranging in size from a few thousands to more than 50,000 worker bees, and the nests are built of wax combs that are partly exposed or placed within cavities such as tree hollows. Larvae are reared in open cells and are fed progressively by workers. Larvae finish feeding and make cocoons in which they undergo metamorphosis within their cocoons and sealed cells. Used waxen cells within the comb matrix are cleaned and re-utilized. Pollen and nectar or honey may be stored adjacent to brood or elsewhere within nest combs. Colonies are perennial and new colonies form by fission, when the laying queen and a swarm of workers leave to found a new colony.

Meliponini: The stingless bees of the world are widely distributed and tropical, with over 600 species in 46 genera [8]. Two genera, *Lestrimellita* and *Cleptotrigona* are parasites of other meliponine genera. These highly eusocial colonies range in size from several dozen workers to nearly 100,000. Nests are often built within cavities and brood cells are made of secreted wax mixed with plant resins, while some species also add feces, mud and other materials, elsewhere in nests. Larvae are reared in brood cells that are mass provisioned, usually with pollen, cephalic gland secretions and nectar. Brood cells are not reused, but the soft nest material of brood cells, called cerumen, is recycled between nest structures. Colonies reproduce by fission and “directed swarming” by a virgin queen and workers to a prepared nest site.

Bombini: Bees in the genus *Bombus,* with approximately 250 species, are large-bodied and generally live in cooler regions, most in the Holarctic [1]. The non-parasitic *Bombus* are primitively eusocial and generally nest in cavities. The social parasites, subgenus *Psithyrus*, enter the nest of another species and take over the role of the host queen, either through chemical means or by killing the resident. Unlike honey bees and stingless bees, colonies are usually univoltine and annual. Larvae are fed progressively by workers, often using food stored [the pocket makers have food stored in a chamber with multiple larvae] elsewhere in the nest.

Euglossini: The orchid bees are entirely restricted to the American tropics, and they are generally medium to large-bodied bees with exceptionally long tongues [9]. The nesting biology of females incompletely known, but nests are either independent constructs of resin, comprised by groups of brood cells partly exposed on hard substrates, but usually are in cavities, and are built with resin, sometimes mixed with wood fragments, mud or feces [10]. Larvae are reared in mass-provisioned cells, and no food is stored in the nest. Unlike other corbiculate bees, female euglossines tend to be solitary, communal, or weakly social, with no castes. Some species of *Eufriesea* construct cells in aggregations within protected areas, with several to many females using the same location over a period of years, but otherwise have limited social interactions [11]. Group living with diverse social structures is known in some species of *Euglossa* and *Eulaema,* including facultatively eusocial species. Males are regularly collected at artificial chemical baits [9].

COLLETIDAE

Caupolicanini: The tribe Caupolicanini consists of large-bodied bees with bilobed short tongues and is restricted to tropical and subtropical America. The genus *Crawfordapis* occurs in mountainous regions from southern Mexico through western Panama [1]. These are solitary bees with burrows excavated in soil, with branches that lead to isolated cells that are lined with a cellophane-like compound to hold larval semi-liquid provisions of diverse pollen and high nectar content [12].

HALICTIDAE

The family of sweat bees is one of the most diverse and abundant bee taxa with more than 4000 species [1]. The majority of species nest in the soil and others in wood, with considerable diversity in female social behavior and group size [13]. Life histories range from uni- to multi-voltine, and behavior ranges from solitary to various forms of social behavior (e.g., communal, or caste-based), and some taxa are parasitic. For social taxa, all but one species has annual life cycles [14] The vast majority of halictines are ground-nesting species, though some species nest in rotting wood or dead plant stems. Our samples include bees from two tribes of Halictinae: the cosmopolitan tribe Halictini, with >2300 species in more than 22 genera [15], and the primarily Neotropical tribe Augochlorini of 646 species [16].

**S2. Supplemental Methods**

*S2.1. Bee sampling and processing*

The euglossine bees were collected at scent attractants applied to clean paper towels and were exclusively males. Meliponini were taken at nest entrances. Natural colonies were added as encountered. Bombini and Diphaglossini came from a premontane rain forest. *Megalopta* (Halictidae: Augochlorini) were from natural nests in stems. *Lasioglossum* (Halictidae: Halictini) were collected at terrestrial nest entrances. Stingless and honey bees at flowers or natural nests were collected by net. A summary is provided in Supplemental Table 1. Repeated sampling of bees from colonies and perennially active solitary bees from the same location offered the opportunity to collect all together over an extended period that included environmental variation, and to assess temporal stability in social and solitary taxa.

All bees were handled, stored and processed using the same protocol. Our insect nets were sterilized with 10% bleach and dried between each site visit to minimize risk of moving contaminants among sites, as has occurred with other pathogens of wildlife [17, 18]. Additional detergents would likely improve best practices for decontaminations protocols that could and perhaps should pertain to many insect-collection protocols to reduce cross contamination between localities and samples [19, 20]. Using sterile nitrile gloves, small bees were collected directly into 2 ml screw cap tubes, preloaded with 100% ethanol. Mid-sized or large bees were stored in 7 or 15 ml in 20- or 50-ml tubes. DNA samples were stabilized on ice within 2 hours or transferred to a portable freezer. Ethanol dispatched bees instantly reduced struggling and metabolic change that could affect their microbiome.

*S2.2. DNA extraction*

For DNA extraction the bee abdomen and hind legs were separated from the rest of the body so that size variation was minimized, DNA extraction kits were not overloaded by flight muscles, and pigments or other potential DNA inhibitors in the compound eyes were absent. The abdominal extract includes most gut microbial cells and diversity [21]. Dissected guts were compared to the surface sterilized abdomen and hind legs (N=6 per treatment group, N=12 per species) for 6 genera and 18 species (Supplemental Table 2). Dissections were done with forceps using blades dipped in 100% ethanol and flame sterilized. We detect little difference between the two treatment types and acknowledge that surface sterilization of bees with 100% ethanol likely removed transient microbes and visually reduced surface pollen and mites — when observed at the time of DNA extraction.

*Sequencing and sample processing*

All samples were submitted for 16S (V3-V4) amplicon sequencing with the Illumina HiSeq 2500 platform at The Hubbard Center for Genome Studies (HCGS), University of New Hampshire. Demultiplexing at HCGS employed bcml2fastq v2.20 and sequencing runs imported into Qiime2 v2021.4. Sequences were denoised using DADA2 in qiime2 then merged [22]. To trim sequences in Qiime2 we used (--p-trim-left-f 19 --p-trim-left-r 20 --p-trunc-len-f 245 --p-trunc-len-r 205). Assigning microbe taxonomy employed classify-consensus-vsearch [23] referencing SILVA 138full-length 99%16S sequences [24, 25], after which sequences that matched chloroplast, mitochondria, Eukaryote and unassigned (bacteria) were removed. We constructed a midpoint-rooted phylogenetic tree using Mafft and Fasttree in QIIME2 align-to-tree-mafft-fasttree pipelinerooted with QIIME2 midpoint-root [26, 27]. Several samples produced moderate host amplification, and bacterial sequences that corresponded to bees were identified using Blast and removed from the 16S dataset. Four reads were on target but were sequenced with the forward index on the reverse primer and the reverse index on the forward primer; these were removed before analyses. Except for three controls (3/19), where sequences present in the extraction blanks were removed from samples, DNA extraction-control samples returned little to no amplification and were excluded from downstream analyses.

*S2.3. Data parsing into testing and training datasets*

To accomplish data parsing the samples were split into a test set and a training set; most samples were in sets of 6 individuals of a species at a location. We assigned the first two samples of each group of 6, species collected at a location at a particular sampling event, as test data and the remainder as training. Songbird trains a null model and a multinomial model on the training data for each set of metadata explored, then predicts and tests this model against the test dataset.

Within our sampling, we did not have sufficient replication of locations within forest types to fully address the influence of forest type on bee microbiomes. Furthermore, only four bee species were well replicated across both locations and forest types, and Songbird revealed little to no unique features across locations. Therefore, we decided to exclude ‘forest type’, and to use ‘location’ as a random effect in our models of seasonal variation in bee microbial richness.

*S2.4. Assigning rank values to morphometric characteristics*

*Tongue length ranged from 1.1mm in Plebeia frontalis*, to 40mm in *Eulaema bombiformis*. We assigned a rank value of (1-4) to accommodate these differences.  *Body length, ranged from 3mm* *Plebeia frontalis to* 26.53mm we assigned a rank value of (1-5) to accommodate these differences. The difference in the range of rank values reflects our confidence in measurements across all bees included in the study. Higher confidence in true values for body length allowed for more rank categories. Body color ranged from light brown to dark black. We assigned a rank value of (1-3) to accommodate these differences. Again, due to the subjective assignment of this body trait, light, we restricted rank values further.

**S3. Supplemental Results**

*S3.1 Predictors of bee microbial richness*

We examined the microbial richness of individual bee species within the two dominant tribes (Euglossini and Meliponini), as well as bee species that we grouped together in an ‘other’ category (Supplemental Table 2). Using observed features (ASVs) as a richness metric, we detected significant variation in microbial richness among the euglossine species (H = 98.91, p = 1.98e-12; richness was highest in *Eufriesea anisochlora* and lowest in *Euglossa cognata*)*.* We found significant variation in the richness of bee microbes for meliponine species (H = 89.06, p = 9.06e-12; richness was highest in *Frieseomelitta nigra* and lowest in *Melipona phenax*), and for bees in the group ‘other’ (H = 21.92, p = 0.0005; richness was highest in *Apis mellifera* and lowest in *Crawfordapis luctuosa* (solitary).

*S3.2. Predictors and structure of bee microbial composition*

We address the principal compositional results in the main text and report these statistics in Supplemental Table 3. However, to further assess the microbial composition of bees by taxonomic and social categories, within each month and thereby removing substantial variability, we present the beta diversity PERMANOVA results for tribe, genus, species and sociality category (Supplemental Table 4). All comparisons (4 variables by 5 months) were significantly different, thus highlight naturally occurring differences between the microbiomes of bee groups.

*S3.3. Location as a predictors of bee microbial composition*

We ran machine learning on location within several species for which we had samples collected across multiple localities (*Euglossa imperialis* (N = 5) *Eugl. crassipunctata* (N=3) *Eugl. mixta* (N=3), *Eulaema nigrita* N=3). Except for *Eugl. imperialis,* model convergence was never reached, and in the case of *Eugl. imperialis* only two features were detected as being significant contributors (*Cutibacterium*, uncultured bacterium, and an unspecified ASV in the family Acetobacteraceae), suggesting that there is limited differentiation in euglossine bee microbiomes within species by location, or there are insufficient data to resolve potential compositional differences. No further investigation of microbial diversity within species by location was warranted.

*S3.4. Lowland tropical forest sites sub-analysis: richness and eveness of* Euglossini and Meliponini

We compared the bacterial richness of Euglossini and Meliponini across 10 months (Supplemental Figure 4a), and richness was significantly higher in Meliponini for each month (September 2018 - W = 10, p-value = 0.000154, November 2018 - W = 6, p-value = 8.814e-05, January 2019 - W = 267, p-value = 0.01118, March 2019 - W = 65, p-value = 4.497e-08 and May 2019 - W = 385, p-value = 0.0002833. Using this same approach, we compared the Pielou evenness of the microbiota between these two tribes by month (Supplemental Figure 4b) and found that evenness was higher in Meliponini for each month, except for November, which is peak wet season (September 2018 - W = 21, p-value = 0.002272, November 2018 - W = 127, p-value = 0.1127, January 2019 - W = 174, p-value = 0.0001283, March 2019 - W = 78, p-value = 5.055e-07, and May 2019 - W = 311, p-value = 1.306e-05). An identical pattern of microbial richness by tribe across months was found using a rarefaction depth of 3,500 per sample (see Rmarkdown in Dryad Folder).

Next, we compared the shared ‘core’ microbiota for both tribes at 5% increments and observed constantly higher numbers of ‘core’ ASVs in Meliponini at each increment. A linear regression with 95% confidence intervals shows this pattern (Supplemental Figure 4C). For our best sampled month, May, euglossine bees and meliponine bees shared 245 ASVs. The percent overlap was 43.6% for euglossine bees and 16.5% for meliponine bees (Supplemental Figure 4D).

*S3.5. Seasonality as an explanatory variable of bee microbial diversity*

Bacterial taxa (ASVs) that were seasonally constrained and assessed by DESeq2 are available in their entirety in the Dryad repository (within Seasonal Bee Microbes).

*S3.6. Bee morphometrics as explanatory variables of bee microbial diversity*

We further examined the contribution of body metrics to microbial richness in the Euglossini and Meliponini separately. First, within Euglossini, again we found rank tongue length to be the best predictor of microbial richness (Supplemental figure 5). Our most informative model, based on Akaike Information Criterion, excluded rank body length and rank body color; [Formula: log(faith_pd) ~ 1 + RANK.TL + RANK.BL + (1 | Genus_species:Location)with rank tongue length (RANK.TL) and rank body length (RANK.BL) as a fixed effect and species withing location as random effects. Significant differences in microbial richness were found for euglossine bees (rank tongue length – Chi-square = 6.6289, df = 1, p = 0.03882); rank body length – Chi-square = 8.2796, df = 2, p = 0.01593.

Using model selection within Meliponini, we found rank body color was the most informative predictor (Supplemental figure 6). This time, the most informative model excluded rank tongue length and rank body length; [ Formula: log(faith_pd) ~ 1 + RANK.COL + (1 | Genus_species:Location) ] with rank color (RANK.COL) as a fixed effect and species within location as random effects. Significant differences were found for Meliponini bees (rank body color – Chi-square =10.259, df = 2, p = 0.00592). The largest differences in microbial richness were found between rank color 1 and 2 (Tukey's - Error =0.13315, z value = -3.126, p =0.00514), and secondarily between rank color 2 and 3 (Tukey's - Error = 0.14425, z value = 2.324, p = 0.05251).

*S3.7. Richness and Composition of CCGM*

We filtered to identify bee samples with CCGM taxa (*Snodgrassella, Gilliamella*, *Lactobacillus and Bifidobacterium*), and identified samples that lacked all CCGM, using the filter --p-no-filter-empty-samples within the Qiime2. We calculated and visualized alpha diversity, and composition as previously described. In Figure S7, we allow for the inclusion of bacterial genus *Frishella*, common amongst bees in the tribe Apis.

*Differential abundance analysis of CCGM in bee species*

Believed to originate from the ileum, there were strains of *Snodgrassella* that had strong positive associations (determined as an association score of 3 or higher) with *Eufriesea pulchra, Euglossa asarophora, Eug. bursigera, Eug. crassipunctata, Eug. mixta, Eulaema meriana, Melipona phenax, M. panamica, M. triplaridis, Partamona musarum, P. peckolti, Scaptotrigona barrocoloradensis, Tetragona ziegleri*, *Tetragonisca angustula, Trigona corvina,* and *T. fulviventris.* Strains of *Gilliamella* had strong association with *Euglossa bursigera, Eug. crassipunctata, Eulaema meriana, Melipona phenax, M. panamica, Partamona musarum, P. peckolti, Tetragona ziegleri, Tetragonisca angustula,* and *Trigona fulviventris*.

Believed to originate from the rectum, there were strains of *Lactobacillus* that had strong positive associations with *Crawfordapis lutcuosa, Eufriesea anisochlora, Euglossa crassipunctata, Eulaema meriana, Lasioglossum umbripenne, Melipona phenax, M. favosa, M. panamica, M. triplaridis, Oxytrigona mellicolor, Partamona musarum, P. peckolti, Scaptotrigona barrocoloradensis, S. luteipennis, S. panamensis, Tetragona ziegleri,* and *Trigona fulviventris.* Strains of *Bifidobacterium* had strong positive associations with *Euglossa asarophora, Eug. crassipunctata, Eulaema meriana, Frieseomelitta nigra, Lasioglossum umbripenne, Melipona panamica, M. triplaridis, Partamona musarum, P. peckolti, Scaptotrigona luteipennis, and Trigona fulviventris*. Within the gut of bees, strong associations exist for diverse strains of CCGM, and we find evidence for substantial specialization between bees and particular strains.

**S4 Supplemental Discussion**

*S4.1. Internal and external comparative microbial analysis*

The lack of differentiation in the microbiome between dissected bee guts and the abdomen suggests that the gut microbiome is the dominant contributor of a bee’s total microbiome, and further suggests dissections are not mandatory to capture the gut microbiome. The lack of differentiation between the two sample types may have been the result of placing bees in 100% ethanol, which would have removed transient microbes. Our results show that our methods were sufficient to study differences of bee gut microbiota.

*S4.2. Limitations of our study*

Within our dataset, we are considering male Euglossini, but we exclusively used females in all other taxa. Catching female euglossine bees in a replicated way is very difficult, and no keys exist for identifying female *Euglossa* to species, or the other non-obligate parasitic genera [28]. For nearly all bee species, the gut microbiome of males and females has not been directly compared. After thorough investigation, we find limited evidence for sex differences in bee gut microbiomes in general. For the best studied bee species *Apis mellifera*, some evidence exists that male hindgut microbiota differ from their female counterparts [29], but how well this finding extends to solitary and facultatively eusocial bees is unknown. A study of *Xylocopa sonoria* (which contains both solitary and facultatively eusocial bees co-occurring in the same landscape), showed microbial composition differed between males and females. The differences between species (*Xylocopa sonoria* and *Xylocopa tabaniformis*) in this study were, however, far greater [30]. Furthermore, the differences in microbial richness between sexes may have resulted from comparing foraging females to newly emerged adult males. Limited sample size also limits the strength of this comparison. Asynchrony in the adult emergence of males and foraging females is a limitation of comparative microbial studies between sexes for most bees.

Another limitation was colony level replication for stingless bees across sites, and thus across forest types. For many species, bee nests were difficult to locate and highly heterogeneous in space. We gradually added new species and localities through the course of the year, and in several instances, we observed colonies disappear. Additionally, a consequence of placing bees in ethanol is that location effects could be erased. Transient microbes which may differentiate one location from another may have been missed. Therefore, we were unable to robustly assess absolute species richness across localities for bee taxa.

*S4.3. Color as an explanatory variable of bee microbial diversity*

We do not observe a clear biological significance to body’s color impact on bee microbiomes. While we consider microbial diversity in the context of color, we did not test this hypothesis here and therefore have insufficient data to determine its relevance, if any. However, if color influences the foraging of bees through sun exposure and thermoregulation. as described in *Cephalotrigona species* [31] and how a bee forages impacts its microbiomes, then color may indirectly impact microbial diversity of bees.

References:

1. Michener CD. The bees of the world. 2000. JHU press.

2. Danforth BN, Minckley RL, Neff JL, Fawcett F. The solitary bees: biology, evolution, conservation. 2019. Princeton University Press.

3. Wcislo WT, Cane JH. Floral resource utilization by solitary bees (Hymenoptera: Apoidea) and exploitation of their stored foods by natural enemies. *Annual review of entomology* 1996; **41**: 257–286.

4. Croat TB. Flora of barro colorado island. 1978. Stanford University Press.

5. Wright SJ, Calderon O. Phylogenetic patterns among tropical flowering phenologies. *Journal of Ecology* 1995; 937–948.

6. Gilbert GS, Reynolds DR. Nocturnal fungi: Airborne spores in the canopy and understory of a tropical rain forest 1. *Biotropica: The Journal of Biology and Conservation* 2005; **37**: 462–464.

7. Grüter C. Stingless Bees. *Cham, Switzerland: Springer International Publishing* 2020.

8. Roubik DW. Stingless bee (Apidae: Apinae: Meliponini) ecology. *Annual Review of Entomology* 2022; **68**: 2023.

9. Roubik DW, Hanson PE. Abejas de orquídeas de la América tropical: Biología y guía de campo. 2004. Editorial INBio.

10. Wcislo DO, Vargas G, Ihle KE, Wcislo WT. Nest construction behavior by the orchid bee Euglossa hyacinthina. *Journal of Hymenoptera Research* 2012; **29**: 15–20.

11. Galgani-Barraza P, Moreno JE, Lobo S, Tribaldos W, Roubik DW, Wcislo WT. Flower use by late nineteenth-century orchid bees (Eufriesea surinamensis, Hymenoptera, Apidae) nesting in the Catedral Basílica Santa María la Antigua de Panamá. *Journal of Hymenoptera Research* 2019; **74**: 65.

12. Roubik DW, Michener CD. Nesting biology of Crawfordapis in Panama (Hymenoptera, Colletidae). *Journal of the Kansas Entomological Society* 1984; 662–671.

13. Wcislo W, Fewell JH, Rubenstein DR, Abbot P. Sociality in bees. *Comparative social evolution* 2017; 50–83.

14. Packer L. The evolution of social behavior and nest architecture in sweat bees of the subgenus Evylaeus (Hymenoptera: Halictidae): a phylogenetic approach. *Behavioral Ecology and sociobiology* 1991; **29**: 153–160.

15. Pesenko YA. The phylogeny and classification of the tribe Halictini, with special reference to the Halictus genus-group (Hymenoptera: Halictidae). *Zoosystematica Rossica* 2004; **13**: 83–113.

16. Gonçalves G. Herd immunity: recent uses in vaccine assessment. *Expert Review of Vaccines* 2008; **7**: 1493–1506.

17. Skerratt LF, Berger L, Speare R, Cashins SD, McDonald KR, Phillott AD, et al. Spread of chytridiomycosis has caused the rapid global decline and extinction of frogs. *EcoHealth* 2007; **4**: 125–134.

18. Foley J, Clifford D, Castle K, Cryan P, Ostfeld RS. Investigating and managing the rapid emergence of white‐nose syndrome, a novel, fatal, infectious disease of hibernating bats. *Conservation biology* 2011; **25**: 223–231.

19. Webb R, Mendez D, Berger L, Speare R. Additional disinfectants effective against the amphibian chytrid fungus Batrachochytrium dendrobatidis. *Diseases of aquatic organisms* 2007; **74**: 13–16.

20. Shelley V, Kaiser S, Shelley E, Williams T, Kramer M, Haman K, et al. Evaluation of strategies for the decontamination of equipment for Geomyces destructans, the causative agent of white-nose syndrome (WNS). *J Cave Karst Stud* 2013; **75**: 1–10.

21. Kwong WK, Moran NA. Evolution of host specialization in gut microbes: the bee gut as a model. *Gut microbes* 2015; **6**: 214–220.

22. Callahan BJ, McMurdie PJ, Rosen MJ, Han AW, Johnson AJA, Holmes SP. DADA2: high-resolution sample inference from Illumina amplicon data. *Nature methods* 2016; **13**: 581–583.

23. Rognes T, Flouri T, Nichols B, Quince C, Mahé F. VSEARCH: a versatile open source tool for metagenomics. *PeerJ* 2016; **4**: e2584.

24. Quast C, Pruesse E, Yilmaz P, Gerken J, Schweer T, Yarza P, et al. The SILVA ribosomal RNA gene database project: improved data processing and web-based tools. *Nucleic acids research* 2012; **41**: D590–D596.

25. Yilmaz P, Parfrey LW, Yarza P, Gerken J, Pruesse E, Quast C, et al. The SILVA and “all-species living tree project (LTP)” taxonomic frameworks. *Nucleic acids research* 2014; **42**: D643–D648.

26. Katoh K, Misawa K, Kuma K, Miyata T. MAFFT: a novel method for rapid multiple sequence alignment based on fast Fourier transform. *Nucleic acids research* 2002; **30**: 3059–3066.

27. Price MN, Dehal PS, Arkin AP. FastTree 2 - Approximately maximum-likelihood trees for large alignments. *PLoS ONE* 2010; **5**: e9490–e9490.

28. Roubik DW. Population traits and a female perspective for Aglae and Exaerete, tropical bee parasites (Hymenoptera, Apinae: Euglossini). *Psyche* 2019; **2019**.

29. Kapheim KM, Rao VD, Yeoman CJ, Wilson BA, White BA, Goldenfeld N, et al. Caste-specific differences in hindgut microbial communities of honey bees (Apis mellifera). *PloS one* 2015; **10**: e0123911.

30. Handy M, Sbardellati D, Yu M, Saleh N, Ostwald M, Vannette R. Incipiently social carpenter bees (Xylocopa) host distinctive gut bacterial communities and display geographic structure as revealed by full-length 16S sequencing. *Authorea Preprints* 2022.

31. Pereboom J, Biesmeijer J. Thermal constraints for stingless bee foragers: the importance of body size and coloration. *Oecologia* 2003; **137**: 42–50.

Supplemental Figures

Supplemental Figure 1


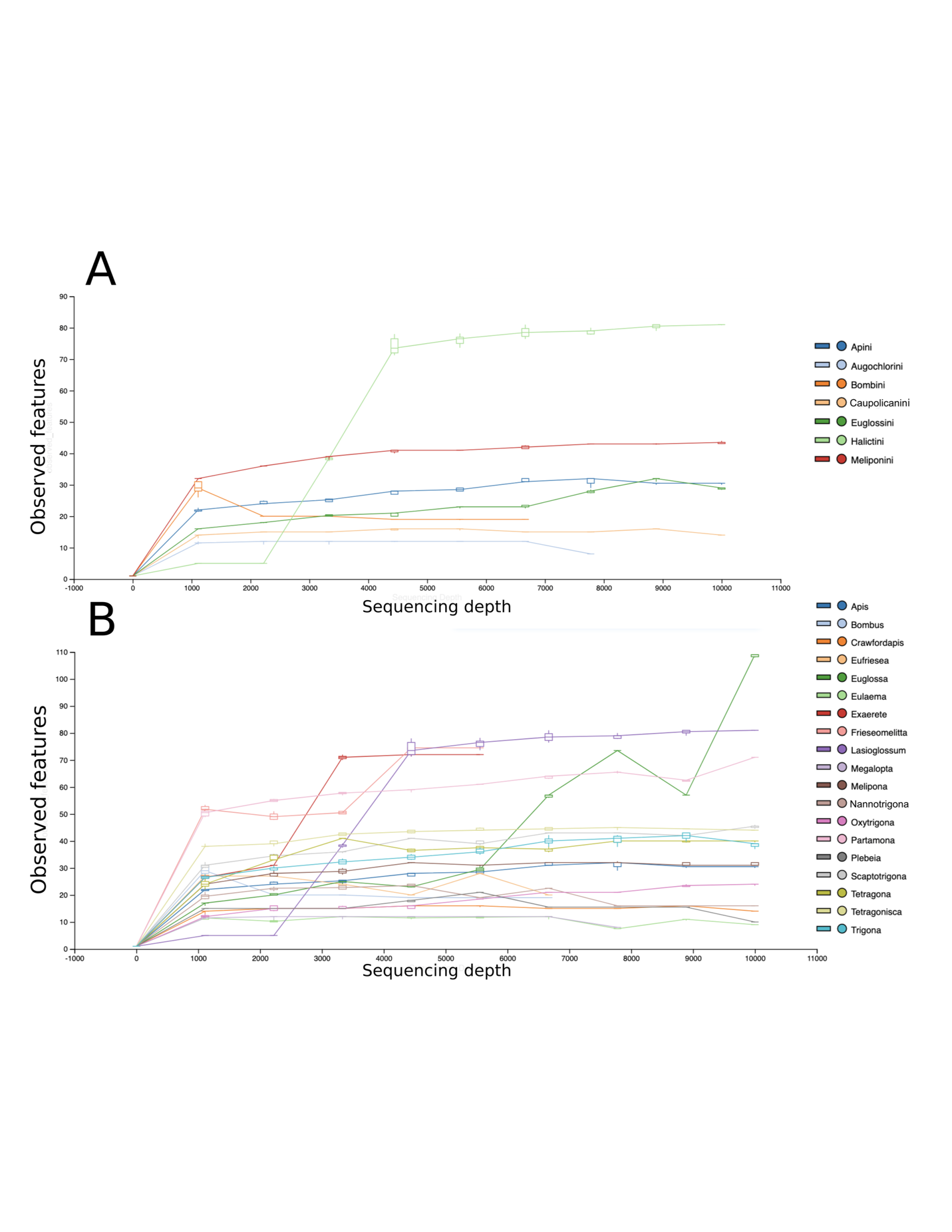


Supplemental Figure 1) Alpha rarefaction curves show observed features found across sequencing depth (0-10,000). A) shows rarefaction curves for 7 bee Tribes. B) shows rarefaction curves for 19 bee genera.

Supplemental Figure 2


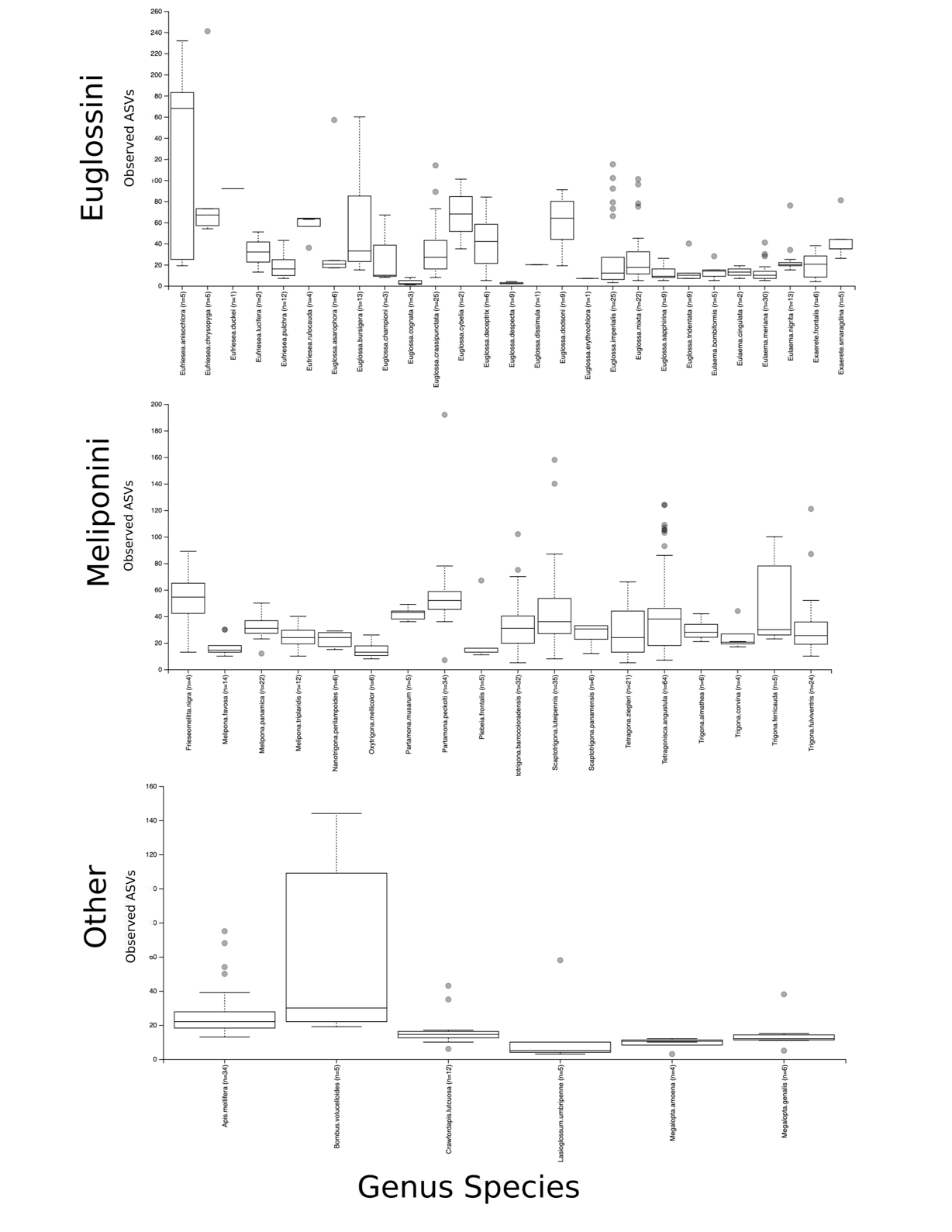


Supplemental Figure 2) Shows Faith's phylogenetic diversity of bee species arranged by tribe (Tribe Euglossini (N=20), Tribe Meliponini (N=18), and Other (N=6). Bee species vary in their bacterial diversity, and we find significant differences within tribes (Kruskal Wallis: Eugossini, H = 98.905, p = 1.976e-12; Meliponini, H = 89.056, p = 9.060e-12; Other, H = 21.9157 p = 0.0005).

Supplemental Figure 3


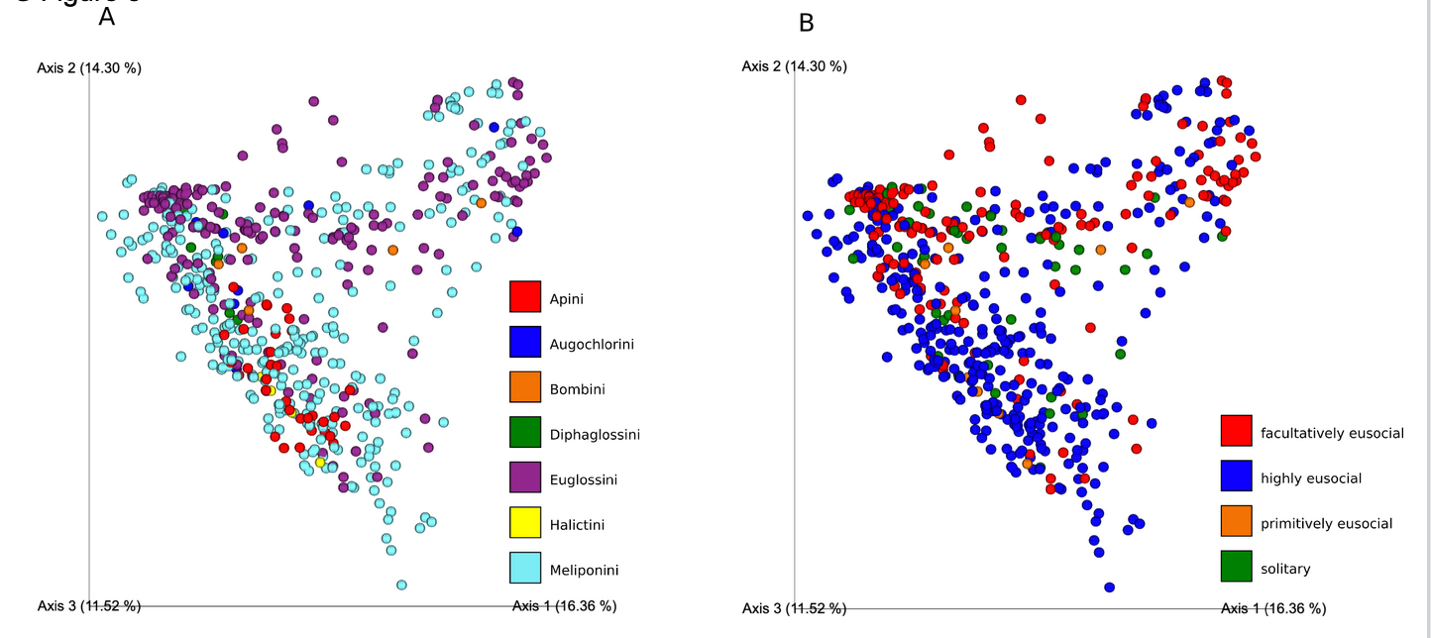


Supplemental Figure 3) A) shows beta diversity (weighted unifrac) of bee samples colored by bee tribe. Significant differences between groups are detected (PERMANOVA; F = 9.497, p < 0.001). B) shows beta diversity (weighted unifrac) of bee samples colored by social category. Significant differences between groups are detected (PERMANOVA; F = 11.165, p < 0.001).

Supplemental Figure 4


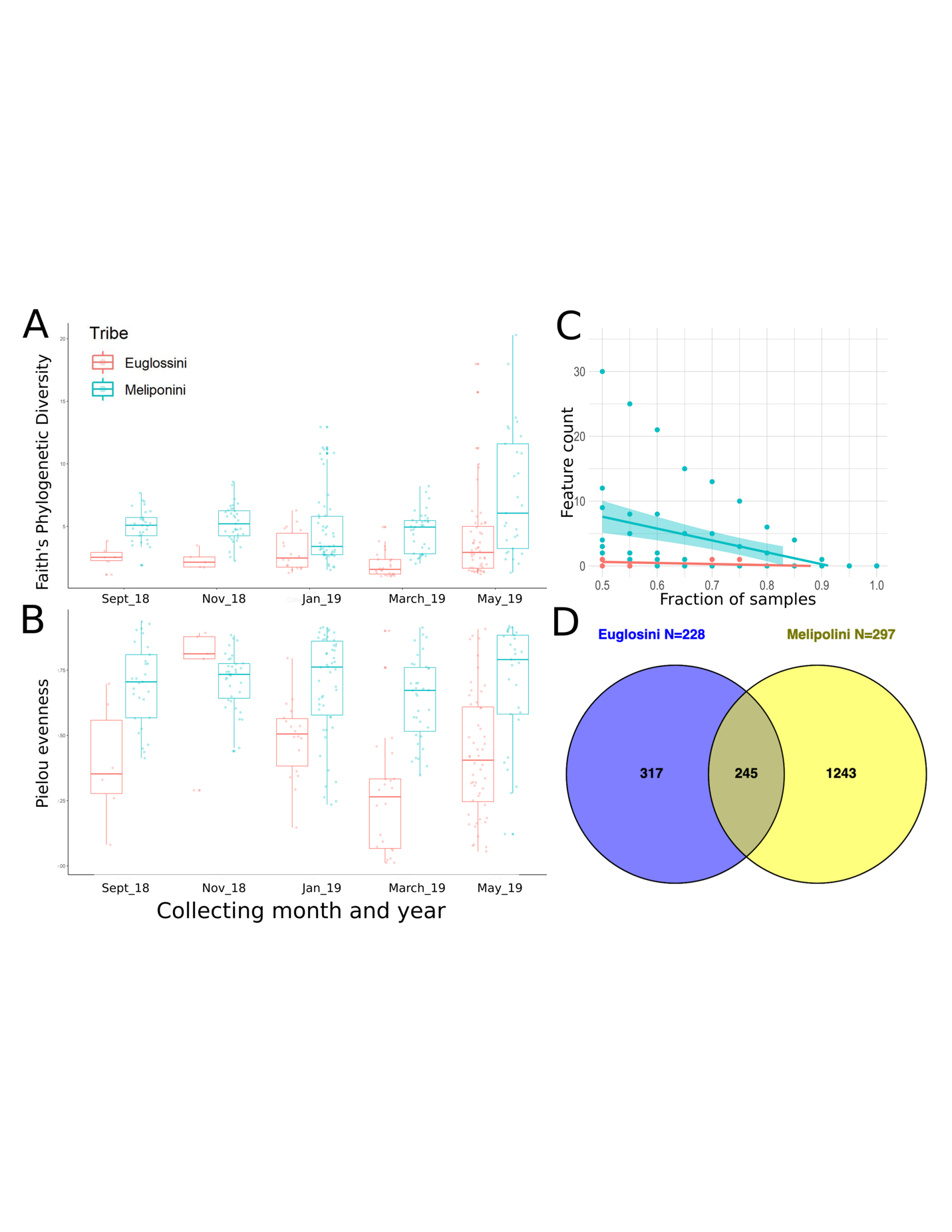


Supplemental Figure 4) A) Summarizes the microbiome for Euglossini and Meliponini collected in lowland tropical forest sites. A) Compares Faiths phylogenetic diversity of Euglossini and Meliponini bees by collection month. Statistical differences between groups were found for each comparison within month (summarized in the main text). B) shows the microbial evenness of Euglossini and Meliponini. Statistical differences between groups were found for each comparison within month, except November (summarized in the main text). C) Core microbial regression shows consistently higher numbers of core taxa for melipinone (highly eusocial) bees compared to Euglossini (facultatively eusocial). D) Venn diagram depicting unique bacterial taxa found in both Euglossini and Meliponini and the overlap between the two groups.

Supplemental Figure 5


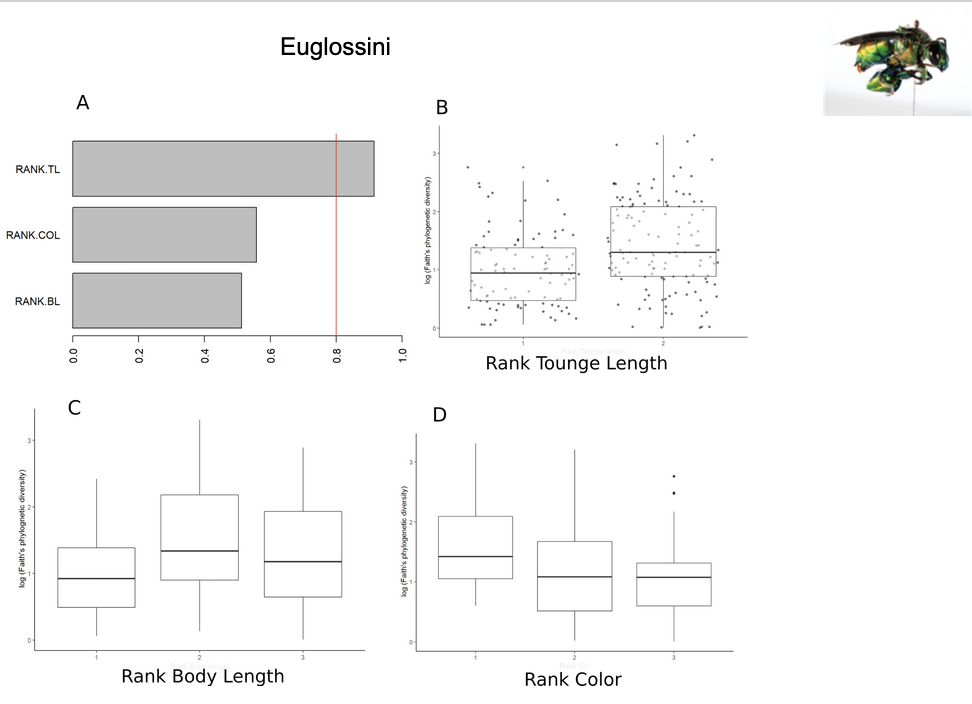


Supplemental Figure 5) shows bee morphometric testing for euglossine bee samples. A) shows model average importance of terms using correlation testing (cor.test; TL = Tongue Length, COL = Color, BL = Body Length). Rank tongue length is the trait that best predicts microbial richness amongst Euglossine bees. B) shows rank tongue length (1 is the longest and 2 is the shortest; significant differences are detected). C) shows rank body length (1 is the longest and 3 is the shortest; significant differences are detected). D) shows rank color (1 is the darkest and 3 is the lightest; no significant differences are detected).

Supplemental Figure 6)


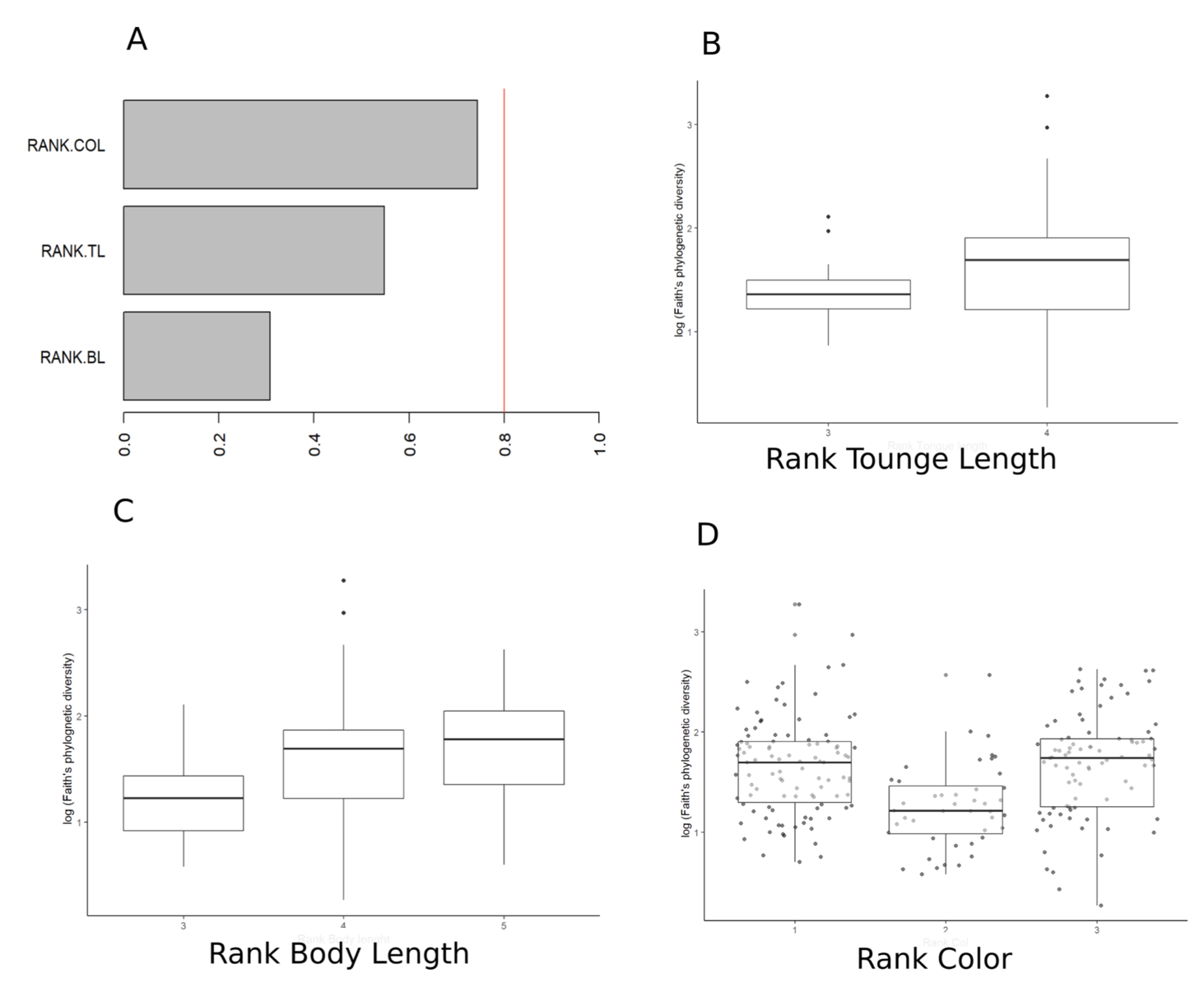


Supplemental Figure 6) shows bee morphometric testing for meliponine bee samples. A) shows model average importance of terms using correlation testing (cor.test; COL = Color, TL = Tongue Length, BL = Body Length). Rank color length is the trait that best predicts microbial richness amongst meliponine bees. B) shows Faith’s phylogenetic diversity arranged by rank tongue length (3 is the longest and 4 is the shortest; no significant differences are detected. C) shows Faith’s phylogenetic diversity of bee species arranged by body length (3 is the longest and 5 is the shortest; no significant differences are detected. D) shows Faith’s phylogenetic diversity arranged by rank color (1 is the darkest and 3 is the lightest; significant differences are detected.

Figure 7


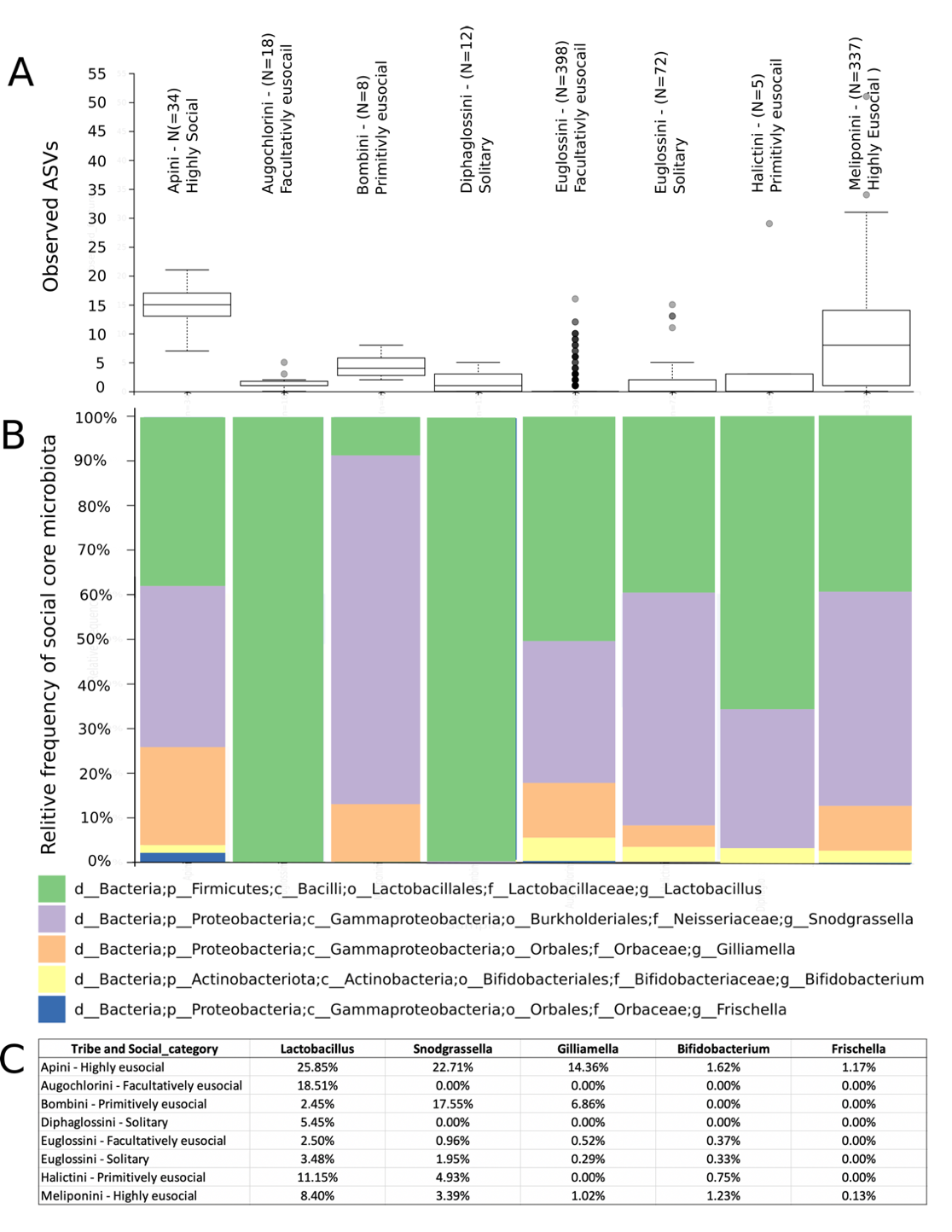


Figure S7) A) Depicts the distributions of CCGM and bacterial genera *Frishella.* A) Boxplots showing the number unique taxa matching to CCGM summarized by tribe and social category. B) Proportional abundance of CCGM only summarized by tribe. C) The percentage of Social Core Microbiome out of all sequences by tribe and by social category. Frishella, a dominant microbial taxa found in the bee genus *Apis*, shows minimal horizontal transmission to Meliponini, and no horizontal transmission to other bee tribes.

Supplemental Figure 8


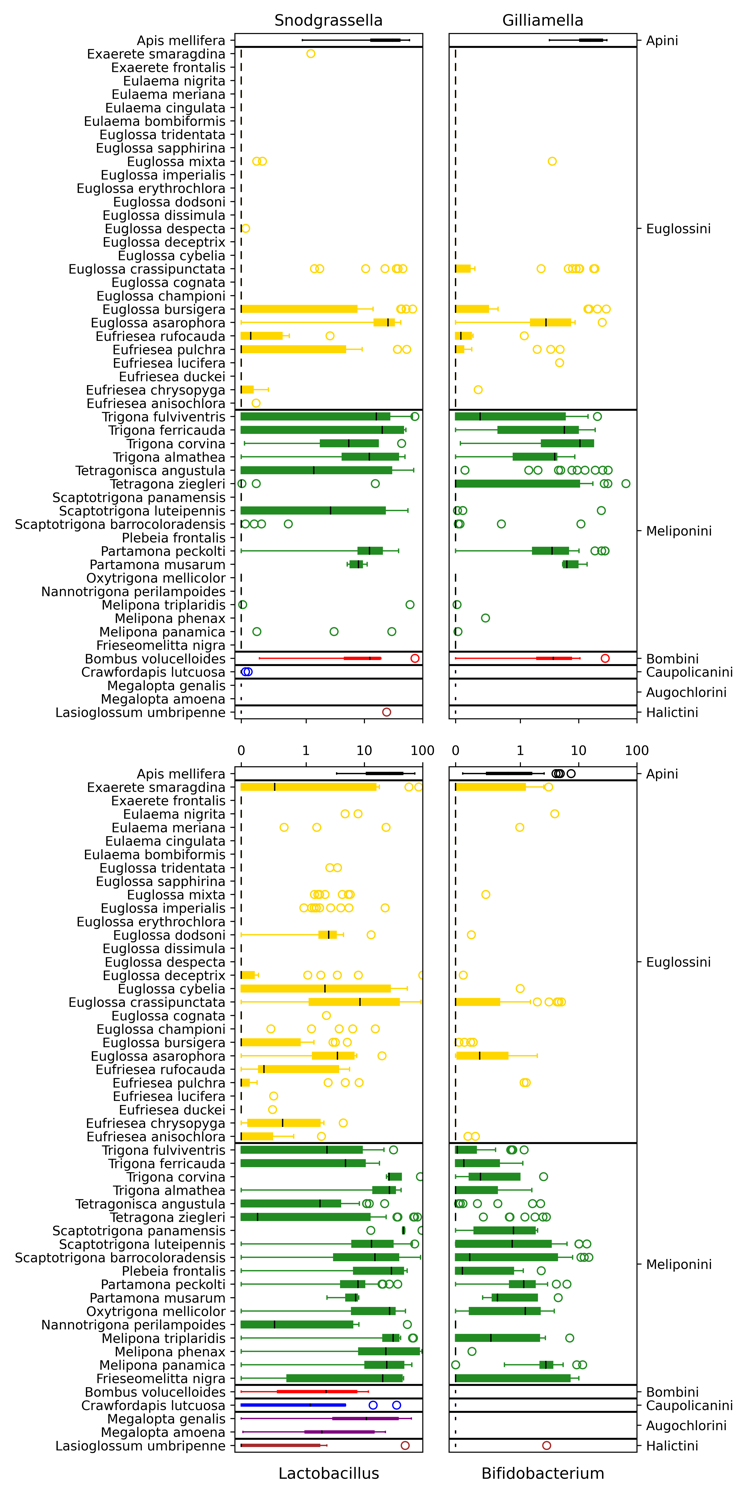


Figure S8) Plots the CCGM and their distributions (percent relative abundance per sample) for all bee species. Boxplots are color coded by bee tribes.

Supplemental Figure 9


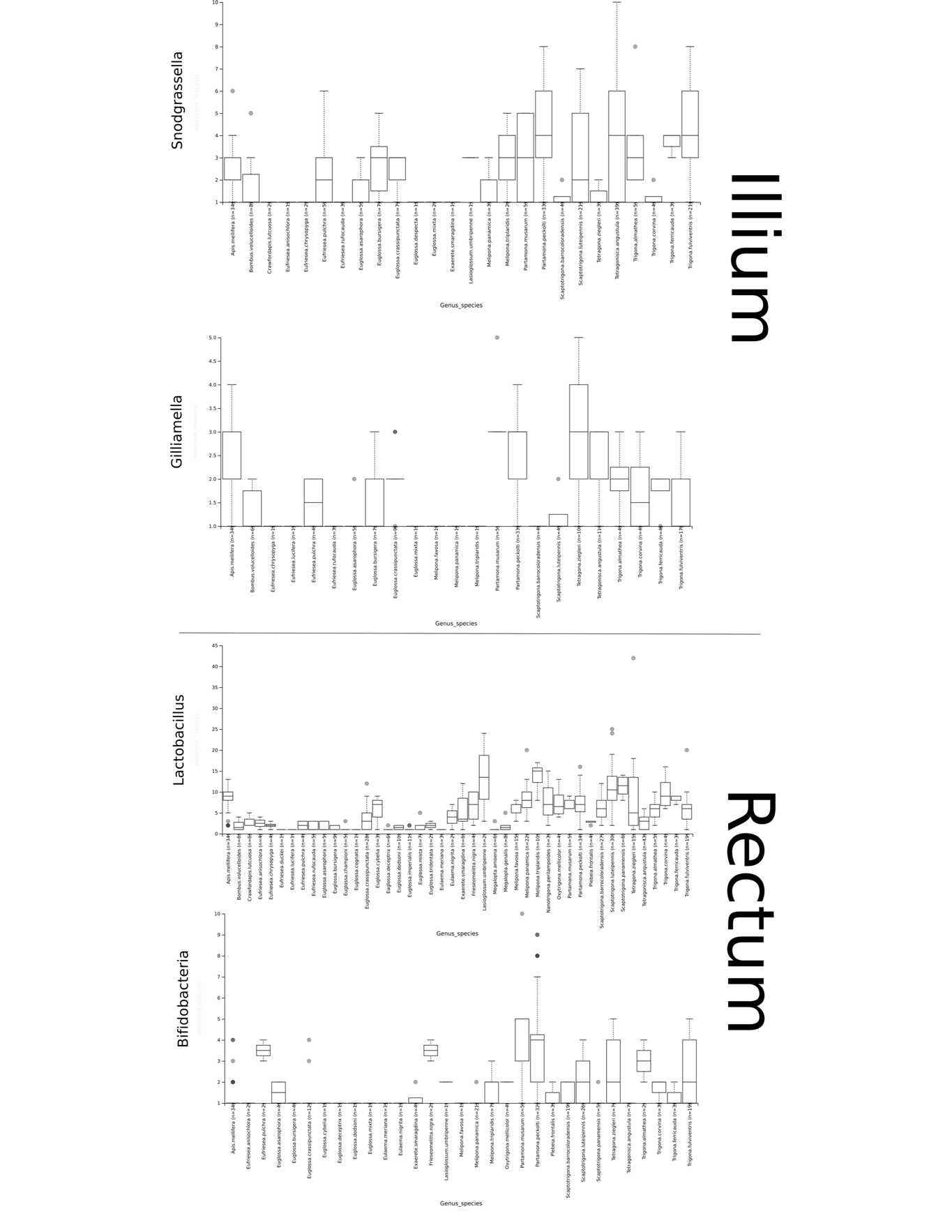


Figure S9, shows the richness of Snodgrassella, Gilliamella, Lactobacillus, and Bifidobacteria ‘’ by bee species.

Supplemental Tables

Supplemental Table 1

Table 1) Table of samples that passed quality control checks. Species are summarized by sampling location. Also shown are the groups that comprise the gut vs. metasoma test (supplemental table 2), the direct seasonal comparison made in Figure 4, the species/location (N > 3) analyses and species that fell below an N=3 after rarefaction.

Supplemental Table 2


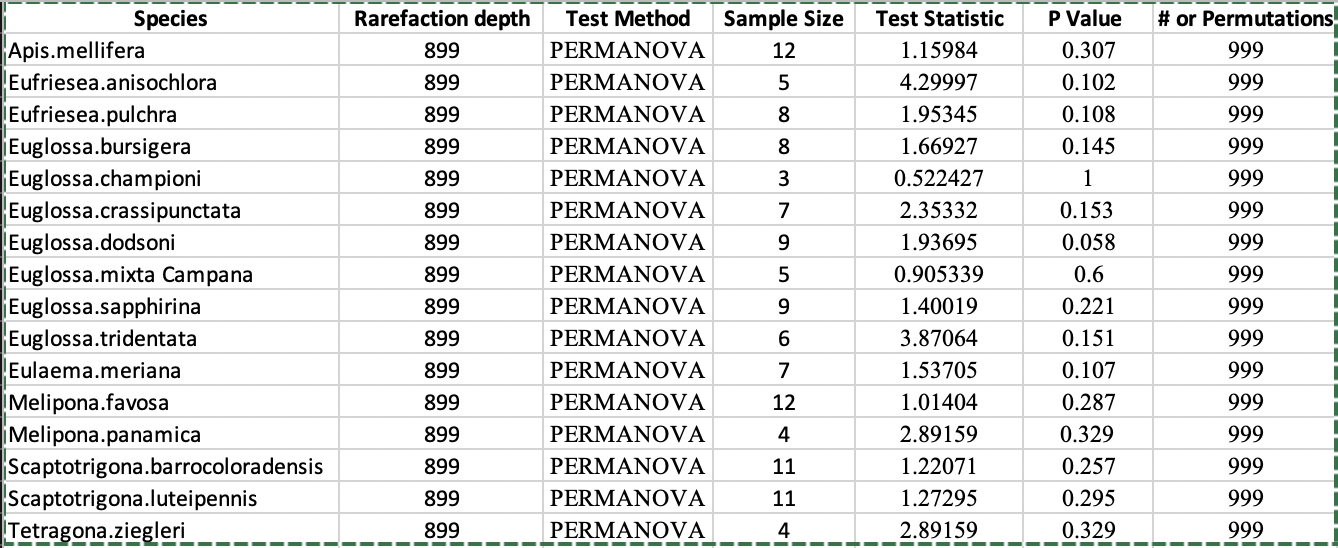


Supplemental Table 2) Compares the microbiome of dissected guts, to the metasoma and hind legs for 19 species, comprising 7 genera. Six individual samples of each type (metasoma and hind legs) were analyzed. Sixteen species that retained sufficient sampling depths (3 were excluded from the original 19) were all non-significant (Weighted Unifrac). Suggesting the major contribution of a bee’s bacterial community comes from its gut, and that the metasoma and hind legs is a viable way to study a bee’s microbiome without needing to undergo dissections.

Supplemental Table 3


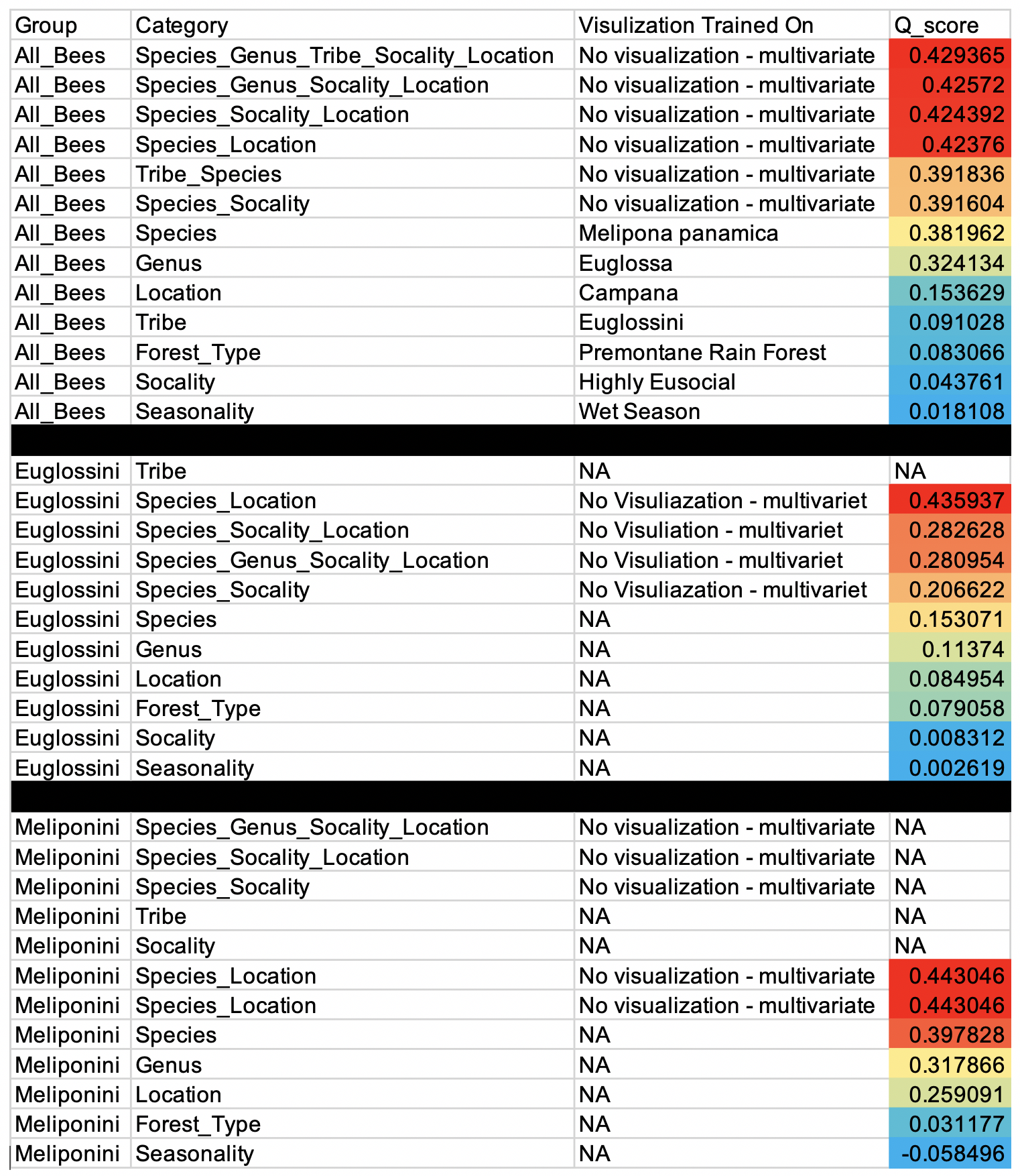


Table 3) Results of multidimensional scaling of the test performance of a Songbird differential abundance model trained on individual bee and environmental factors: sociality category, tribe, genus, species, forest type, location, season. The Q score shows the percentage that the compositionally aware model outperformed a trivial model.

Supplemental Table 4


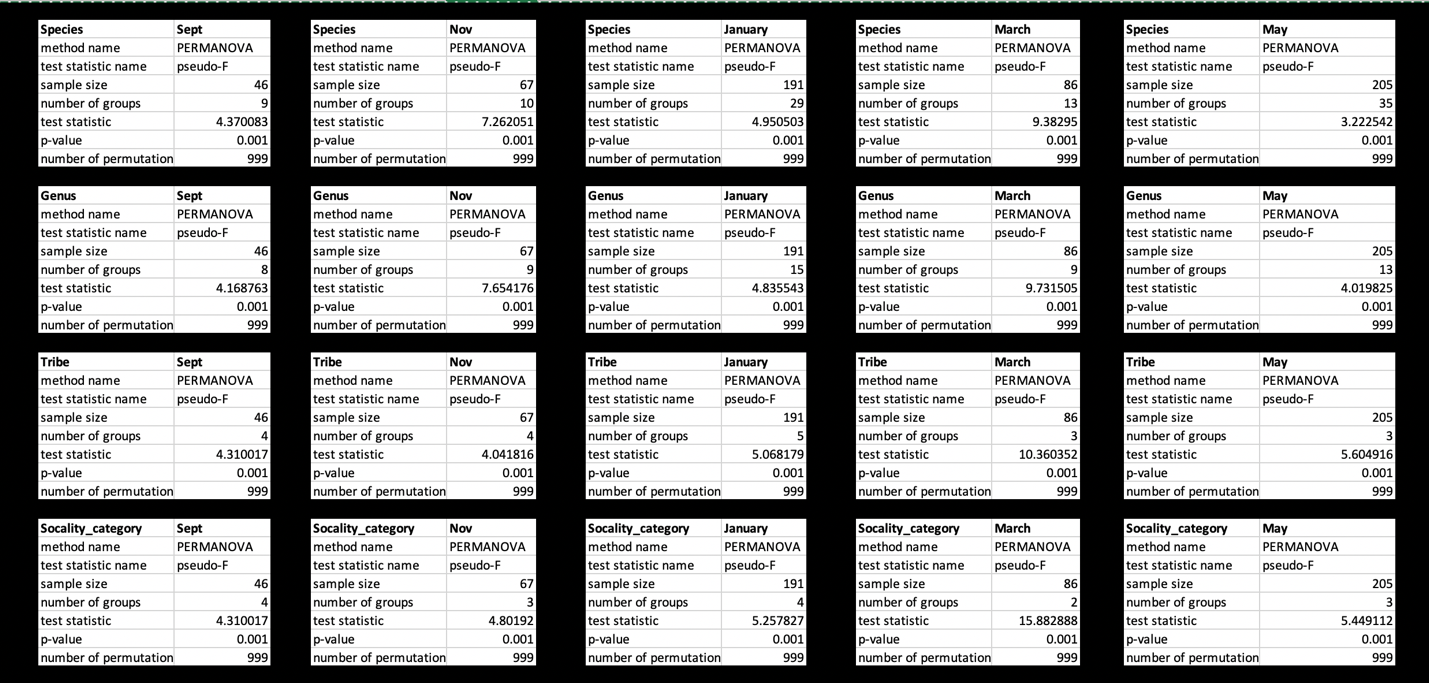


Supplemental Table 4) Summary statistics of (weighted unifrac) beta diversity of bee species, bee genera, bee tribes and bee sociality (Tribe Euglossini (N=20), Tribe Meliponini (N=18), and Other (N=6) that were collected and compared within month from Sept 2018 until May 2019. All levels of comparisons of bee groups across all months show significant differentiation p < 0.001.
